# *Chlamydia trachomatis* Co-opts GBF1 and CERT to Acquire Host Sphingomyelin for Distinct Roles during Intracellular Development

**DOI:** 10.1371/journal.ppat.1002198

**Published:** 2011-09-01

**Authors:** Cherilyn A. Elwell, Shaobo Jiang, Jung Hwa Kim, Albert Lee, Torsten Wittmann, Kentaro Hanada, Paul Melancon, Joanne N. Engel

**Affiliations:** 1 Department of Medicine, University of California, San Francisco, California, United States of America; 2 Microbial Pathogenesis and Host Defense Program, University of California, San Francisco, California, United States of America; 3 Microbiology and Immunology, University of California, San Francisco, California, United States of America; 4 Cell and Tissue Biology, University of California, San Francisco, California, United States of America; 5 Department of Biochemistry and Cell Biology, National Institute of Infectious Disease, Tokyo, Japan; 6 Department of Cell Biology, University of Alberta, Edmonton, Canada; Duke University, United States of America

## Abstract

The obligate intracellular pathogen *Chlamydia trachomatis* replicates within a membrane-bound inclusion that acquires host sphingomyelin (SM), a process that is essential for replication as well as inclusion biogenesis. Previous studies demonstrate that SM is acquired by a Brefeldin A (BFA)-sensitive vesicular trafficking pathway, although paradoxically, this pathway is dispensable for bacterial replication. This finding suggests that other lipid transport mechanisms are involved in the acquisition of host SM. In this work, we interrogated the role of specific components of BFA-sensitive and BFA-insensitive lipid trafficking pathways to define their contribution in SM acquisition during infection. We found that *C. trachomatis* hijacks components of both vesicular and non-vesicular lipid trafficking pathways for SM acquisition but that the SM obtained from these separate pathways is being utilized by the pathogen in different ways. We show that *C. trachomatis* selectively co-opts only one of the three known BFA targets, GBF1, a regulator of Arf1-dependent vesicular trafficking within the early secretory pathway for vesicle-mediated SM acquisition. The Arf1/GBF1-dependent pathway of SM acquisition is essential for inclusion membrane growth and stability but is not required for bacterial replication. In contrast, we show that *C. trachomatis* co-opts CERT, a lipid transfer protein that is a key component in non-vesicular ER to *trans*-Golgi trafficking of ceramide (the precursor for SM), for *C. trachomatis* replication. We demonstrate that *C. trachomatis* recruits CERT, its ER binding partner, VAP-A, and SM synthases, SMS1 and SMS2, to the inclusion and propose that these proteins establish an on-site SM biosynthetic factory at or near the inclusion. We hypothesize that SM acquired by CERT-dependent transport of ceramide and subsequent conversion to SM is necessary for *C. trachomatis* replication whereas SM acquired by the GBF1-dependent pathway is essential for inclusion growth and stability. Our results reveal a novel mechanism by which an intracellular pathogen redirects SM biosynthesis to its replicative niche.

## Author Summary

*C. trachomatis* is the leading cause of non-congenital blindness in developing countries and is the number one cause of sexually transmitted disease and non-congenital infertility in Western countries. The capacity of *Chlamydia* infections to lead to infertility and blindness, their association with chronic diseases, and the extraordinary prevalence and array of these infections make them public concerns of primary importance. This pathogen must establish a protective membrane-bound niche and acquire essential lipids from the host cell during infection in order to survive and replicate. This study identifies novel mechanisms by which *C. trachomatis* hijacks various lipid trafficking proteins for distinct roles during intracellular development. Disruption of these lipid trafficking pathways results in alterations in the growth and stability of its protective niche as well as a defect in replication. Understanding the molecular mechanisms of these host-pathogen interactions will lead to rational approaches for the development of novel therapeutics, diagnostics, and preventative strategies.

## Introduction

*Chlamydia* species are obligate intracellular pathogens that cause a wide range of diseases in humans, including sexually transmitted, ocular, and respiratory tract infections [1]. The capacity of *Chlamydia* infections to lead to infertility and blindness, their association with chronic diseases such as atherosclerosis, and the extraordinary prevalence and array of these infections make them public concerns of primary importance [1], [2].

All *Chlamydia* species share a dimorphic developmental cycle that allows them to survive within the hostile environment of the host cell (reviewed in [3]). *Chlamydia* alternate between an extracellular, spore-like infectious form termed the elementary body (EB), and an intracellular, metabolically active but non-infectious form termed the reticulate body (RB). Infection is initiated by binding of the EB to the host cell where it is taken up by an actin and Rho family GTPase-dependent process and sequestered within a unique membrane bound compartment called the inclusion [4]. Subsequently, the EB differentiates into an RB and replicates by binary fission within the inclusion. Concomitantly, the bacteria begin remodeling the inclusion membrane by insertion of bacterial proteins that promote segregation of the inclusion from the classical endosomal/lysosomal transport pathway, that facilitate interactions of the inclusion with the exocytic transport pathway, and that promote migration of the inclusion along microtubules to the peri-Golgi region [5], [6]. The developing inclusion expands to accommodate increasing numbers of bacteria and is stabilized by recruitment of host cytoskeletal structures primarily composed of F-actin and intermediate filaments [7]. After 24–72 hours (hrs) of replication, RBs redifferentiate back to EBs and are released from the host cells by cell lysis or active extrusion [8].

*Chlamydiae* are one of the few known bacterial pathogens that require host-derived membrane lipids, including sphingomyelin (SM) and cholesterol, for intracellular growth and development [6], [9], [10], [11], [12], [13], [14]. Recent work suggests that SM biosynthesis is also required for homotypic fusion of multiple inclusions within the same cell as well as for inclusion membrane stability [15]. *Chlamydia* are thought to acquire SM by vesicular trafficking via multiple routes, including (1) the interception of SM-containing Golgi-derived exocytic vesicles destined for the plasma membrane (PM) [16], [17], [18], (2) fusion with multivesicular body (MVB)-derived vesicles [15], [19], [20], and/or (3) Golgi fragmentation [21], [22]. SM acquisition by the inclusion is observed as early as 2 hrs post infection (hpi), with incorporation of SM into the inclusion membrane and into the bacterial cell wall [6], [10], [12], [13], [15], [19], [20], [23]. Golgi fragmentation induced by *C. trachomatis* has been shown to play an important role in SM acquisition during the later stages of infection and may be required for subsequent fusion with exocytic vesicles by a mechanism that involves Rab6 and Rab11 [21], [22]. SM transport to the inclusion is partially abrogated by Brefeldin A (BFA), an inhibitor of vesicular transport [6], [24], [25] and is accompanied by a decrease in inclusion size [6], indicating that SM transport to the inclusion via vesicular trafficking is important for inclusion growth. Importantly, BFA treatment has no effect on bacterial replication [6], suggesting that SM acquisition by a BFA-sensitive pathway is not essential for replication and that *C. trachomatis* may acquire SM by additional routes that involve BFA-insensitive and/or non-vesicular trafficking pathways.

ADP ribosylation factors (Arfs) are small GTPases that are key players in the regulation of vesicular transport where they function to recruit coat proteins necessary for vesicle formation (reviewed in [26]). Arf1 is localized in all three Golgi compartments, *cis-, medial-, and trans*-Golgi, and cycles between an active GTP bound form and an inactive GDP-bound form [27]. Arf1 activation is spatially and temporally regulated by specific guanine nucleotide exchange factors (GEFs), GBF1 (Golgi-specific BFA resistance guanine nucleotide exchange factor 1) and BIG1/BIG2 (BFA-inhibited guanine nucleotide exchange proteins 1 and 2) which are present in the *cis*- and *tran*s-Golgi, respectively [26], [28], [29], [30], [31], [32], [33]. BFA inhibits the activation of Arf1 by targeting GBF1 and BIGs [29], [34], [35], [36]. GBF1 is required for the assembly and maintenance of the Golgi stack whereas BIGs are required for maintenance of the *trans*-Golgi network [33], [37]. Besides regulating coat protein recruitment, Arf1 controls the actin cytoskeleton by modulating the lipid microenvironment via recruitment of various phosphoinositide kinases [38], and it also regulates the vimentin architecture in cells [39]. Although Arf1 localizes to the inclusion and has recently been implicated in *Chlamydia* infection [40], [41], the specific roles of Arf1 and its *cis*- and *trans*-Golgi-specific GEFS in inclusion biogenesis and/or acquisition of SM has not been previously investigated.

Until recently, vesicular trafficking of ceramide from the ER to the *trans*-Golgi was believed to be essential for SM biosynthesis; however, it is now appreciated that the major pathway of *de novo* SM biosynthesis involves a cytosolic lipid transfer protein, called CERT, that transports ceramide (the precursor for SM) from the ER directly to the *trans*-Golgi, where it is converted to SM by one of two SM synthases, SMS1 or SMS2 [42], [43], [44], [45], [46]. CERT has three functional domains, an N-terminal PH (pleckstrin homology) domain that binds phosphoinositide-4-phosphate (PI4P) at the *trans*-Golgi, a FFAT (two phenylalanines in an acidic tract) motif that binds integral membrane proteins, VAP-A and VAP-B, at the ER, and a C-terminal START (steroidogenic acute regulatory protein-related lipid transfer) domain that binds to and extracts ceramide from the ER membrane [47]. CERT is subjected to regulation within a serine repeat (SR) domain where phosphorylation by Protein Kinase D and hyperphosphorylation by Casein Kinase Iγ2 (CKIγ2) inactivates CERT while dephosphorylation by Protein Phosphatase 2Cε activates CERT [48], [49], [50]. Transfer of ceramide from the ER to the Golgi via CERT is thought to occur at ER-Golgi membrane contact sites (MCS), sub-regions of the ER located very close to *trans*-Golgi stacks [51], [52]. The role of non-vesicular lipid transport pathways during *Chlamydia* infection has not been previously explored.

In this study, we examined the role of specific host proteins required for vesicular and non-vesicular lipid transport during infection to define their contribution to SM acquisition, bacterial replication, and inclusion biogenesis. We found that *C. trachomatis* selectively co-opts GBF1 within the *cis*-Golgi compartment for vesicle-mediated SM acquisition and that this source of SM contributes to inclusion membrane growth and stability but that GBF1 function is not required for bacterial replication. Unexpectedly, we discovered that *C. trachomatis* coordinates recruitment of CERT and SM synthases to the inclusion and that subversion of this lipid synthesis pathway is required for bacterial replication and for efficient SM acquisition by *C. trachomatis*. We propose that *C. trachomatis* utilizes separate lipid trafficking pathways to acquire SM for distinct roles during infection: SM acquired by the GBF1-dependent pathway is essential for inclusion growth and stability while CERT-dependent transport of ceramide and subsequent conversion to SM is necessary for *C. trachomatis* replication.

## Results

### GBF1 but not BIG1/2 function is partially required for SM acquisition but dispensable for *C. trachomatis* replication

In an RNA interference (RNAi) screen for host factors required for *C. trachomatis* L2 infection of *Drosophila* S2 cells, homologs of Arf1 and its associated GEFs, GBF1 (localized to the *cis*-Golgi) and BIG1 (localized to the *trans*-Golgi) were identified [53]. Since *Chlamydia* is thought to occupy a post Golgi compartment and since *C. trachomatis* induced Golgi fragmentation enhances replication [6], [21], [22], we tested the hypothesis that spatially-localized Arf1 function within the Golgi might be important for SM acquisition and/or for *C. trachomatis* intracellular growth.

**Figure 1:**
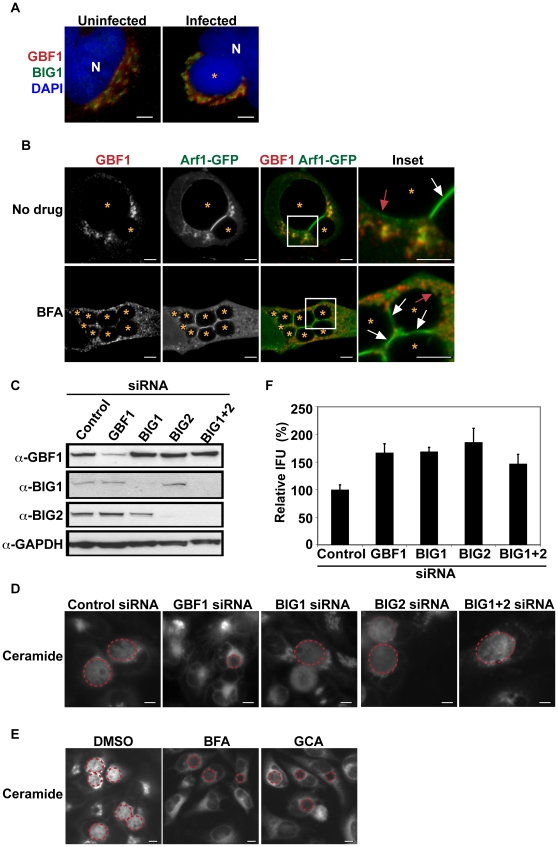
GBF1 function is required for SM acquisition but not for *C. trachomatis* replication. (A) HeLa cells were infected with *C. trachomatis* L2 for 24 hrs and then fixed and stained with antibodies to GBF1 (red) and BIG1 (green). Bacteria and host DNA were detected using DAPI (blue). The *cis* and *trans* polarity of the Golgi was maintained in *C. trachomatis* L2-infected cells. N, host nucleus. *, inclusion. Scale bar = 5 µm. (B) HeLa cells were transfected with Arf1-GFP for 18 hrs, infected with *C. trachomatis* L2 for 24 hrs in the absence or presence of 10 µM BFA, and then fixed and stained with antibodies to GBF1 (red). Enlargements of boxed regions are shown to the right. Images represent a single z slice from confocal images. The exposure time for each filter set for all images was identical. Arf1-GFP localized to the region between two closely apposed inclusions (white arrow) and to a thin rim around the inclusion (red arrow) whereas GBF1 was excluded from these regions. *, inclusion. Scale bar = 5 µm. (C) Western blot analysis of siRNA-treated samples. GAPDH was used as a loading control. (D) HeLa cells were depleted of GBF1, BIG1, and/or BIG2 for 3 days, infected with *C. trachomatis* L2 for 24 hrs, and then labeled with BODIPY FL-Ceramide to visualize SM acquisition by the inclusion. The exposure time for all images was identical. Dashed red lines demarcate the inclusions. Scale bar = 5 µm. (E) HeLa cells were infected with *C. trachomatis* for 24 hrs, treated with 10 µM BFA or GCA during the last 3 hrs of infection, and then labeled with BODIPY FL-Ceramide to analyze SM acquisition by the inclusion. The exposure time for all images was identical. Dashed red lines demarcate the inclusions. Scale bar = 5 µm. (F) HeLa cells were depleted of GBF1, BIG1, and/or BIG2 for 3 days, infected with *C. trachomatis* L2 for 24 hrs, and then analyzed for progeny formation as described in Materials and Methods. Values (mean ± standard error) are shown as percentage of control siRNA-treated samples. No significant decrease in progeny formation was observed. IFU, inclusion forming units.

We first examined the localization of Arf1-GFP, GBF1, and BIG1 during *C. trachomatis* infection of HeLa cells. We found that GBF1 and BIG1 were localized to the fragmented Golgi around the inclusion in *C. trachomatis*-infected cells and appeared to maintain their *cis* and *trans* polarity, as observed in uninfected cells (Figure 1A). GBF1 (Figure 1B) and BIG1 (data not shown) were primarily localized adjacent to but not on the inclusion membrane. Similar results were obtained in cells expressing GFP-tagged GBF1 (data not shown). In contrast, Arf1-GFP was found not only on Golgi vesicles adjacent to the inclusion but was also localized to the inclusion membrane, where it formed a thin rim of circumferential staining (Figure 1B), consistent with previously published results [40], [41]. We noted a striking concentration of Arf1-GFP at the region of two closely apposed inclusion membranes (Figure 1B). To evaluate whether GBF1 or BIG1/2 activity was required for Arf1-GFP recruitment to the inclusion membrane, we treated cells with BFA, which inhibits both GBF1 and BIGs, and examined the localization Arf1-GFP and GBF1. Exposure of the cells to BFA resulted in dispersion of GBF1 and Arf1-GFP throughout the cell and loss of the circumferential rim staining on the inclusion; however, BFA had no effect on Arf1-GFP localization at abutting inclusion membranes (Figure 1B). This finding suggests that GBF1 and/or BIG1/2 activities are required for Arf1 recruitment to the outer region of the inclusion but that they are not necessary for Arf1 maintenance at closely apposed inclusions.

To determine the specific contribution of the *cis*- versus *trans*-Golgi compartments to SM acquisition, HeLa cells were depleted of GBF1 or BIG1/2 by siRNA, infected with *C. trachomatis*, and then examined by live-cell microscopy for SM acquisition as described in the Materials and Methods. Protein depletion was assessed by western blot analysis (Figure 1C). As shown in Figure 1D, labeled lipids were readily visible inside bacterial inclusions in control siRNA-treated cells and in cells depleted of BIG1 and/or BIG2. In contrast, depletion of GBF1 resulted in ∼60% reduction in SM acquisition by the inclusion (Figures 1D and S1A; p<0.001). SM acquisition was similarly reduced in cells treated with Golgicide A (GCA), a GBF1-specific inhibitor [37] and with BFA ([17] and Figures 1E and S1B). As GCA targets GBF1 selectively and does not inhibit BIGS, these results suggest that the relevant target of BFA during *C. trachomatis* L2 infection is GBF1.

We next tested whether GBF1 or BIG1/2 were required for intracellular growth. GBF1 depletion (Figure S1B) or inhibition with GCA or BFA (Figure S2) resulted in inclusions that were smaller than those observed in control siRNA- or untreated cells. Quantitation of inclusion size, as described in Materials and Methods, revealed that the average inclusion size in GBF1-depleted cells was decreased by 40% (p<0.001) compared to control siRNA-treated cells (Figure S1D). In contrast, depletion of BIG1 and/or BIG2 gave rise to inclusions that were slightly larger than those observed in control siRNA depleted cells (Figures S1C and S1D). Despite the changes in inclusion size, depletion of any of the Arf1 GEFS did not decrease production of progeny; in fact, a small increase in progeny was observed (Figure 1F). The reason for the increase in progeny is not currently known.

Together, these results demonstrate that GBF1, but not BIG1/2 function, is required for vesicle-mediated SM acquisition and inclusion growth but is dispensable for bacterial replication. These findings indicate that the impact of disrupting GBF1 function on SM acquisition is not an indirect result of altering Arf1-dependent trafficking in the *trans*-Golgi, since depletion of BIG1 and/or BIG2 did not reduce SM acquisition or inclusion growth. We note that in *Drosophila* S2 cells, depletion of either GBF1 or the single BIG1 was sufficient to decrease inclusion formation [53]; this finding may reflect differences in Golgi organization between *Drosophila* S2 and mammalian cells, and/or it may reflect the fact that these proteins may have additional roles beyond the secretory pathway in host cells.

### GBF1 function is required for inclusion membrane stability

**Figure 2:**
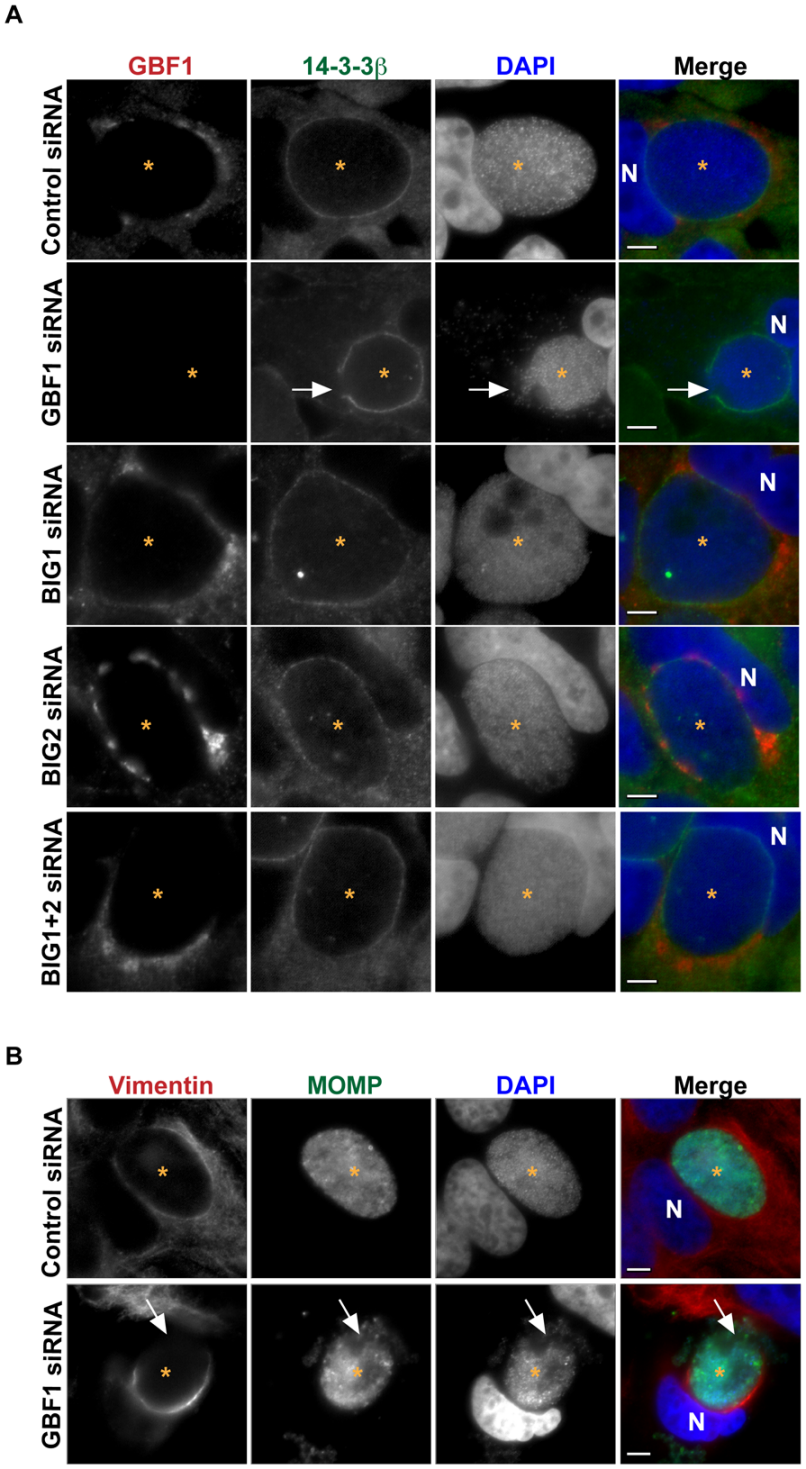
GBF1 but not BIG1/2 function is required for inclusion membrane stability. (A) HeLa cells were treated with the indicated siRNA for 3 days, infected with *C. trachomatis* L2 for 24 hrs, then fixed and stained with antibodies to 14-3-3β (green) to identify the inclusion membrane and GBF1 (red). Bacteria and host DNA were detected using DAPI (blue). The exposure time for each filter set for all images was identical. White arrows point to breaks in the inclusion membrane where the bacteria are released into cytoplasm in GBF1-depleted cells. Inclusions formed in BIG1 and/or BIG2 depleted cells remain intact. (B) HeLa cells were depleted of GBF1 for 3 days, infected with *C. trachomatis* L2 for 24 hrs, then fixed and stained with antibodies to MOMP (green) to identify bacteria and vimentin (red). Bacteria and host DNA were detected using DAPI (blue). The exposure time for each filter set for all images was identical. White arrows point to the region on the inclusion that is devoid of vimentin staining and where bacteria are released into the cytoplasm. N, host nucleus; *, inclusion. MOMP, *C. trachomatis* major outer membrane protein. Scale bar = 5 µm.

We observed that interference with GBF1 function resulted in a defect in SM acquisition by the inclusion, and recent studies have shown that the inclusion membrane integrity is compromised when host SM biosynthesis is inhibited [15]. Therefore, we tested whether GBF1 plays a role in maintaining the integrity of the inclusion membrane. Depletion of GBF1 (Figure 2A) or exposure to GCA or BFA (Figure S2A) resulted in a loss of inclusion membrane integrity, as evidenced by discontinuities in 14-3-3β staining (which binds to the inclusion membrane protein, IncG) and release of bacteria into the host cytoplasm at the broken membrane sites. Quantitative analysis (as described in Materials and Methods) revealed that ∼80% of infected cells in which GBF1 function was inhibited exhibited broken inclusions (Figures 2 and S2A). The loss of inclusion membrane integrity was observed at mid- to late times of infection (data not shown), after significant replication has occurred.

Recent studies have shown that the inclusion is stabilized by a cage-like structure composed of F-actin and vimentin [7]. Although it has been reported that inhibition of SM biosynthesis did not result in alteration of the vimentin or actin structure around the inclusion [15], experiments using BFA have revealed that perturbations in vesicular trafficking lead to reciprocal changes in the architecture of the vimentin network in the host cell, indicating a tight link between membrane trafficking and the cytoskeleton [39]. Furthermore, Arf1 has been shown to regulate actin dynamics at Golgi membranes [54]. We therefore considered whether the loss of inclusion integrity observed upon disruption of GBF1 function was due solely to the decrease in SM acquisition or whether disruption of GBF1 function also affected the vimentin and actin cage surrounding the inclusion. We examined the localization of these cytoskeletal proteins in infected cells treated with GBF1 siRNA or GCA. Disruption of GBF1 function caused both the vimentin and actin cages to collapse to a thin, hemispheric cup-like structure on the nuclear side of the inclusion (Figures 2B, S2B, and S2C). In cells treated with either BFA or GCA, we also observed less GBF1 staining surrounding the inclusion, and GBF1 displayed a more compact Golgi-like localization (Figure S2A). GBF1 inhibited cells were more sensitive to Triton extraction (data not shown), similar to what has been reported following disruption of the actin and vimentin cage during infection [7], suggesting that the cytoskeletal cage is compromised upon GBF1 inhibition.

Together, these results indicate that GBF1 function contributes to the stability of the inclusion membrane by a mechanism that involves SM acquisition and/or maintenance of the vimentin and F-actin cage around the inclusion; however, GBF1 function is not required for intracellular replication. Given that SM biosynthesis is essential for replication [6], [12], our findings suggest that other lipid transport mechanisms may be involved in the acquisition of host SM.

### The ceramide transport protein, CERT and its ER binding protein, VAP-A, are recruited to the inclusion

**Figure 3:**
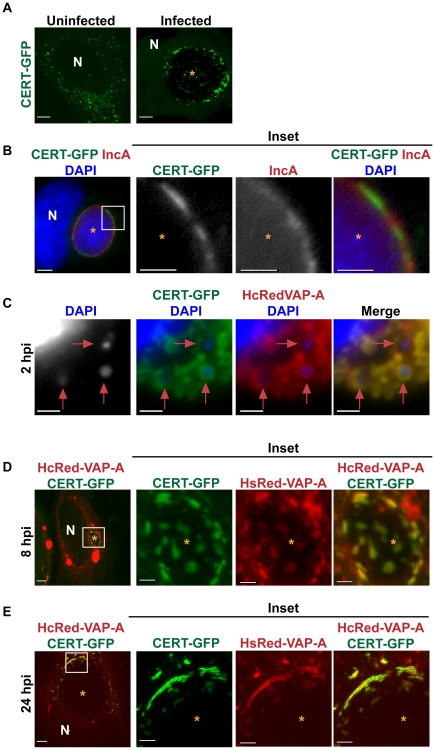
CERT and VAP-A are recruited to the inclusion. (A) HeLa cells transfected with CERT-GFP for 18 hrs were left uninfected or infected with *C. trachomatis* L2 for 24 hrs. Images shown are maximum intensity projections of confocal z-stacks (0.4-µm slices). N, host nucleus; *, inclusions. Scale bar = 5 µm. (B) HeLa cells transfected with CERT-GFP were infected with *C. trachomatis* serovar D for 24 hrs and then fixed and stained with antibodies to IncA (red) to identify the inclusion membrane. Bacteria and host DNA were detected using DAPI (blue). Enlargements (inset) of boxed regions are shown to the right. *, inclusions. Scale bar = 5 µm. (C–E) HeLa cells were transfected with CERT-GFP and HcRedVAP-A for 18 hrs and infected with *C. trachomatis* L2 for (C) 2, (D) 8, or (E) 24 hrs. (C) Cells were stained with DAPI to visualize the nascent inclusions (red arrows). (D and E) Enlargements (inset) of boxed regions are shown to the right. At 8 and 24 hpi, CERT-GFP and HcRedVAP-A colocalize on the inclusion membrane and exhibit a patchy distribution. Images shown are maximum intensity projections of confocal z-stacks (0.4-µm slices). N, host nucleus; *, inclusions. Scale bar = 5 µm, except with insets from panels B, C, and D where scale bar = 2.5 µm.

Since ceramide transport from the ER to the *trans*-Golgi by the cytosolic lipid transporter, CERT, is the predominant pathway for SM biosynthesis [42], [43], [44], [45]), we tested whether CERT-dependent trafficking was involved in SM acquisition by *C. trachomatis*. We first examined the localization of CERT during *C. trachomatis* infection of HeLa cells that were transiently transfected with CERT-GFP. In uninfected cells, CERT-GFP preferentially localized to the Golgi as well as to punctate, vesicle-like structures outside the Golgi (Figure S4 and [45], [55]), which are thought to be ER-derived (K. Kumagai and K. Hanada, personal communication). In *C. trachomatis*-infected cells, CERT-GFP was recruited to the inclusion, exhibiting a non-homogeneous, patchy distribution on the inclusion membrane surface (Figure 3A, B, D, E, Video S1). CERT-GFP appeared on the same plane as but distinct from localization of the *C. trachomatis* inclusion membrane protein, IncA (Figure 3B). This pattern of CERT recruitment to the inclusion was confirmed using an antibody to endogenous CERT (Figure S3), demonstrating that localization was not an artifact of ectopic expression. CERT-GFP also localized to *C. trachomatis* serovar D inclusions (Figure 3B), suggesting that the recruitment to the inclusion was not serovar-specific. CERT-GFP was recruited to inclusions as early as 2 hpi, where nascent inclusions were enveloped by CERT-GFP-containing vesicles that partially overlapped with p58, a marker of the ER-Golgi intermediate compartment (ERGIC) (Figure S4A).

CERT binding to VAP at the ER is required for CERT to extract ceramide from the ER and deliver it to the *trans*-Golgi [45], where ceramide serves as a substrate for SM synthesis by *trans*-Golgi localized SMS1 and SMS2 [46], [56]. Previous work suggests that there is an intimate association of the ER with the chlamydial inclusion [57], [58]. We examined the localization of CERT and VAP-A by co-expressing CERT-GFP and HcRed-VAP-A [45] in infected cells and found that HcRed-VAP-A colocalized with CERT-GFP at the inclusion at 2, 8, and 24 hpi (Figures 3C–E). These results suggest that CERT may function to bring the ER membrane in close apposition to the inclusion or that VAP-A is recruited directly to the inclusion.

### CERT is required for bacterial replication and SM acquisition

Given the proximity of the Golgi and the striking recruitment of CERT to the inclusion, we used three complementary approaches to determine whether CERT plays a role in *C. trachomatis* infection: (1) pharmacological inhibition of CERT by HPA-12, a synthetic analog of ceramide that specifically inhibits CERT-mediated ceramide transfer [59], [60], (2) overexpression of Casein Kinase Iγ2 (CKIγ2), which plays a crucial role in down-regulating CERT activity [48], and (3) depletion of CERT by siRNA.

**Figure 4:**
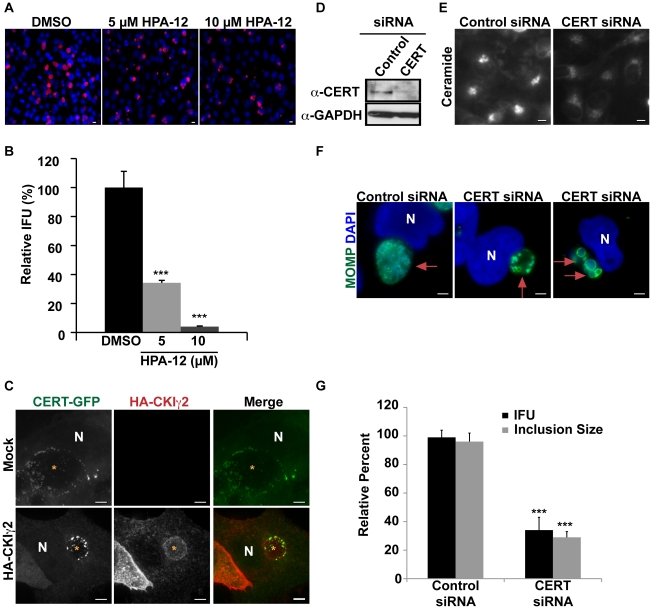
CERT function is required for *C. trachomatis* replication. HeLa cells were infected with *C. trachomatis* L2, treated with the indicated concentration of HPA-12 at 1–24 hpi, and then (A) fixed and stained with antibodies to MOMP (red) and with DAPI (blue) to visualize bacteria or (B) analyzed for progeny formation. Values (mean ± standard error) are shown as percentage of DMSO treated samples. Data are representative of 3 independent experiments. ***p<0.001 compared to DMSO treated cells (ANOVA). (C) HeLa cells were transfected with CERT-GFP and HA-CKIγ2 for 18 hrs, infected with *C. trachomatis* L2 for 24 hrs, and then fixed and stained with antibodies to HA (red). The exposure time for each filter set of all images was identical. Images shown are maximum intensity projections of confocal z-stacks (0.4-µm slices). Ectopic expression of HA-CKIγ2 decreased inclusion size but did not affect CERT-GFP recruitment to the inclusion membrane. Scale bar = 5 µm. (D) Western blot analysis of siRNA-treated samples. GAPDH was used as a loading control. (E) HeLa cells were depleted of CERT for 3 days and then labeled with BODIPY FL-Ceramide to analyze SM accumulation in the Golgi. The exposure time of all images was identical. CERT depletion reduced SM accumulation in the Golgi. Scale bar = 5 µm, (F) HeLa cells were depleted of CERT for 3 days, infected with *C. trachomatis* L2 for 24 hrs, and then fixed and stained with antibodies to MOMP (green) to identify the inclusion. Bacteria and host DNA were detected using DAPI (blue). The exposure time for all images was identical. CERT depletion reduced inclusion size. Red arrows point to inclusions. Scale bar = 5 µm. (G) HeLa cells were depleted of CERT for 3 days, infected with *C. trachomatis* L2 and analyzed for inclusion size and progeny formation. Values (mean ± standard error) are shown as percentage of control siRNA samples. CERT depletion significantly reduced inclusion size and progeny formation. Data are representative of 2 independent experiments. ***p<0.001 for CERT siRNA-treated cells compared to control siRNA-treated cells (ANOVA). N, host nucleus. IFU, inclusion forming units. Scale bar = 5 µm.

We first examined inclusion morphology and progeny production in cells treated with increasing concentrations of HPA-12. Cells were infected for 1 hr and then incubated in the absence or presence of HPA-12 for an additional 23 hrs. We observed a dose-dependent decrease in inclusion formation (Figures 4A) and progeny production (Figure 4B) in cells exposed to HPA-12, resulting in a 96% reduction in progeny at 10 µM HPA-12 compared to DMSO (p<0.001). At the doses employed, HPA-12 did not affect host or bacterial cell viability nor did it affect bacterial binding and entry (data not shown).

CKIγ2 regulates CERT by hyperphosphorylation of a SR motif located within the middle region, which induces an autoinhibitory interaction between the PH and START domains. This event interferes both with the ability of CERT to bind PI4P and with its ability to transport ceramide, though CERT is still able to interact with VAP-A at the ER [48], [61]. Overexpression of CKIγ2 is sufficient to inhibit CERT function and to cause dissociation of CERT from the Golgi complex [61]. In cells co-transfected with CERT-GFP and HA-tagged CKIγ2, we observed a significant defect in inclusion development, with the appearance of small inclusions (Figure 4C), similar to the results observed with HPA-12 (Figures 4A and 6C). Importantly, CERT-GFP was still recruited to the inclusion in cells expressing HA-tagged CKIγ2 (Figure 4C). These results are consistent with the notion that CERT activity is required for inclusion development and suggest that PI4P binding is not required for CERT recruitment to the inclusion.

We next examined inclusion morphology in HeLa cells depleted of CERT for 72 hrs and subsequently infected with *C. trachomatis* for 24 hrs. The efficiency of CERT depletion was determined in uninfected cells by western blot analysis (Figure 4D). Loss of CERT activity was confirmed by demonstrating decreased accumulation of BODIPY FL-SM and BODIPY FL-Ceramide at the Golgi apparatus (Figure 4E). CERT depletion resulted in a ∼66% (p<0.001) reduction in inclusion size as well as a ∼60% (p<0.001) decrease in progeny production (Figures 4F–G). Depletion or inhibition of CERT sometimes resulted in the appearance of inclusions containing aberrant appearing RBs (Figure 4F and data not shown). Progeny formation was decreased more robustly in HPA-12 treated cells than in CERT-depleted cells, most likely because CERT depletion was not complete, although we cannot rule out the possibility that HPA-12 may have additional off-target effects.

**Figure 5:**
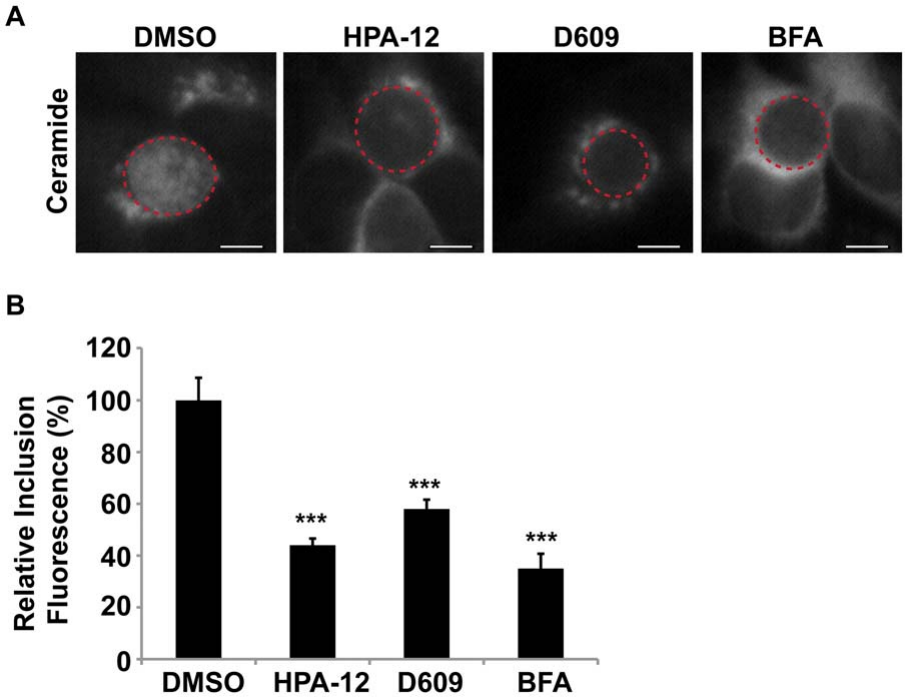
CERT function is required for SM acquisition. (A) HeLa cells were infected with *C. trachomatis* L2 for 24 hrs, treated with 5 µM HPA-12 for the last 3 hrs of infection, and then labeled with BODIPY FL-Ceramide to analyze SM accumulation by the inclusion. As a control for decreased SM acquisition by the inclusion, cells were also treated with 10 µM BFA or 25 µg/ml D609. The exposure time of all images was identical. Dashed red lines demarcate inclusions. The residual fluorescence likely represents Golgi staining. Scale bar = 5 µm. (B) Quantitation of SM acquisition following treatment with HPA-12, D609, or BFA. Values (mean ± standard error) are shown as percentage of mean fluorescence intensities relative to DMSO-treated samples. ***p<0.001 (ANOVA). HPA-12, D609, and BFA-treated cells displayed a significant decrease in fluorescence intensity of the inclusion and its contents compared to DMSO-treated samples.

We next explored whether CERT was required for the SM accumulation in the inclusion by monitoring incorporation of BODIPY FL-Ceramide. Since depletion of CERT led to a significant reduction in inclusion size (Figure 4F), we monitored lipid transport in the presence of the CERT inhibitor, HPA-12. HeLa cells were infected for 20 hrs to allow inclusions to develop to a large enough size for robust visualization by phase microscopy, then exposed to HPA-12 for 3 hrs, and subsequently pulse-labeled with BODIPY FL-Ceramide. The fluorescent lipid readily accumulated in inclusions within DMSO-treated cells, however, inclusions in HPA-12-treated cells showed ∼56% (p<0.001) decrease in fluorescence intensity (Figure 5). Similar results were observed in control cells treated with D609, an inhibitor of SMS1 and SMS2 [62], [63], [64] or with BFA (Figure 5; p<0.001).

Since CERT is localized to the Golgi during infection, it is possible that CERT is indirectly involved in *C. trachomatis* infection based on its role in host SM biosynthesis at the Golgi. In addition or alternatively, as CERT and VAP-A are also localized at the inclusion, CERT may be directly involved in *C. trachomatis* infection by mediating the transfer of a portion of host ceramide from the nearby ER directly to the inclusion. We examined the localization of ceramide in infected cells expressing CERT-GFP using a ceramide antibody. This antibody does not cross-react with SM, cholesterol, or other phospholipids under physiologic *in vivo* conditions [65]. Ceramide appeared to be highly enriched in the region adjacent to the inclusion in what is likely to be the surrounding ER (Figure S5). Importantly, we also found that a fraction of ceramide localized at the inclusion membrane as evidence by co-staining with IncA antibodies and Arf1-GFP (Figure S5A and S5B). Although we found ceramide in close association with CERT-GFP at the inclusion membrane, ceramide and CERT-GFP did not overlap on the inclusion membrane (Figure S5C), possibly reflecting conversion of ceramide to SM and loss of reactivity with the ceramide antibody. Taken together, these results indicate that CERT is required for *C. trachomatis* replication and that interference with CERT correlates with a block in the SM acquisition by the inclusion. In addition, the localization of ceramide during infection suggests that CERT may be functioning at the Golgi as well as at the inclusion.

### CERT transfer activity and ceramide binding are required for its recruitment to the inclusion

To determine the mechanism of CERT recruitment to the inclusion, we examined the consequences of inactivating specific domains of CERT. The ability of CERT to transfer ceramide for SM biosynthesis requires: (1) interaction with the Golgi membrane (PI4P) via its PH domain, (2) an interaction with the ER membrane proteins VAP-A and VAP-B via its FFAT motif, and (3) the ability to bind ceramide via its START domain [47]. We were particularly interested in the PH domain, as recent studies have shown that multiple proteins that regulate PI4P metabolism are recruited to the inclusion, including OCLR (oculocerebrorenal syndrome of Lowe protein 1) and the PI4P kinases, PI4KIIα and to a lesser extent PI4KIIβ [40]. The presence of PI4P at the inclusion combined with our finding that VAP-A colocalizes with CERT at the inclusion (Figures 3C–3E) raises the possibility that CERT could be recruited to the inclusion by its interaction with PI4P and/or with VAP-A.

**Figure 6:**
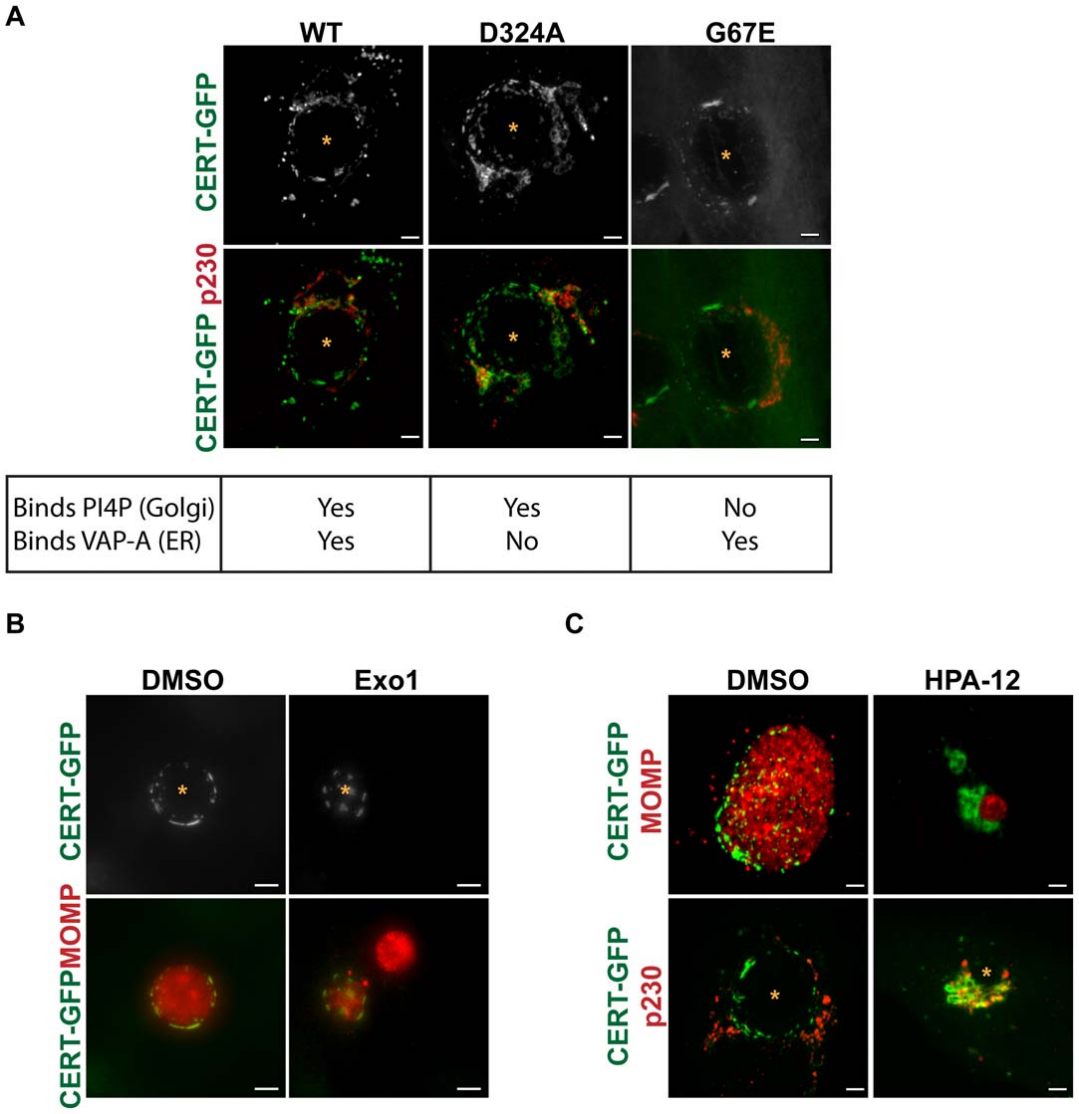
CERT transfer and/or ceramide binding activity are required for its recruitment to inclusions. (A) HeLa cells were transfected with CERT-GFP, CERT (D324A)-GFP, or CERT (G67E)-GFP, infected with *C. trachomatis* L2 for 24 hrs, and then fixed and stained with an antibody to p230 (red) to identify the *trans*-Golgi. The exposure time for each filter set of all images was identical. Images shown are maximum intensity projections of confocal z-stacks (0.4-µm slices). Mutation of the PI4P binding (G67E) or VAP-A binding (D324A) domains did not affect CERT-GFP recruitment to the inclusion. Scale bar = 5 µm. (B) HeLa cells expressing CERT-GFP were infected with *C. trachomatis* L2, treated with 50 µM Exo1 (Arf1 inhibitor) for 1–24 hpi, and then fixed and stained with antibodies to MOMP (red) to identify bacteria. The exposure time for each filter set of all images was identical. Images shown are maximum intensity projections of confocal z-stacks (0.4-µm slices). CERT-GFP localization to the inclusion was unaffected by Arf1 inhibition. Scale bar = 5 µm. (C) HeLa cells expressing CERT-GFP were infected with *C. trachomatis* L2, treated with 5 µM HPA-12 for 1–24 hpi, and then fixed and stained with antibodies to MOMP (red) to identify bacteria or to p230 (red) to identify the *trans*-Golgi. The exposure time for each filter set of all images was identical. Inhibition of CERT transfer and/or ceramide binding activity by HPA-12 treatment resulted in loss of CERT accumulation on the inclusion membrane. Images shown are maximum intensity projections of confocal z-stacks (0.4-µm slices). Scale bar = 5 µm. N, host nucleus; *, inclusion.

We examined the localization of two GFP-tagged CERT mutants: CERT (G67E), which contains a mutation in the PH domain that prevents CERT from binding PI4P at the Golgi, or CERT (D324A), which harbors a mutation in the FFAT motif that prevents CERT from interacting with VAPs at the ER [43], [45] during *C. trachomatis* infection. In uninfected cells, CERT (G67E)-GFP is distributed throughout the cell while CERT (D324A)-GFP is concentrated at the Golgi (data not shown and [45]). As shown in Figure 6A, both CERT (G67E)-GFP and CERT (D324A)-GFP localized to the inclusion at 24 hpi in a manner indistinguishable from CERT-GFP. While CERT-GFP and CERT (D324A)-GFP show some Golgi localization during infection, none is observed for CERT (G67E)-GFP (Figure 6A). Co-staining with antibodies to Calnexin, an ER marker, revealed that although the ER appears in close proximity to the inclusion, it does not overlap with CERT-WT, D324A, or G67E at the inclusion (Figure S6). CERT was still recruited to inclusions formed in a mutant cell line of Chinese hamster ovary cells, LY-A, which carries a mutation in the PH domain of endogenous CERT (G67E) (Figure S3) [43], [66]. Despite the fact that LY-A cells have reduced SM biosynthesis [43], [66], SM acquisition by *C. trachomatis* inclusions was normal in these cells (data not shown).

We next tested whether Arf1 activity was required for CERT recruitment, since Arf1 localizes to the inclusion and since the PH domains of some lipid transfer proteins, such as FAPPs (four-phosphate-adaptor protein 2) and OSBP (oxysterol binding protein), can simultaneously bind PI4P and Arf1 [67]. *C. trachomatis*-infected HeLa cells were treated with Exo1, a specific inhibitor of Arf GTPase activity [68], from 1 to 24 hpi, and CERT-GFP localization was examined at 24 hpi. Although quantitation of inclusion size in Exo1 treated cells revealed a ∼64% decrease (p<0.001), Exo1 had no effect on CERT recruitment to the inclusion (Figure 6B). Together, these results indicate that interaction with PI4P, VAP-A, or Arf1 alone is not essential for CERT recruitment to the inclusion, though PI4P, VAP-A, or Arf1 binding may function redundantly or there may be other determinants within CERT. In addition, CERT-GFP was still recruited to inclusions in cells treated with GCA, BFA, or Nocodazole, which disrupts microtubules (Figures S4B and 7C), indicating that GBF1 function, vesicular trafficking, and microtubules are not required for CERT recruitment.

To test whether CERT lipid transfer activity and/or ceramide binding is required for CERT recruitment to the inclusion, we treated cells expressing CERT-GFP with 5 µM HPA-12 from 1 to 24 hpi. In control experiments, HPA-12 treatment did not affect CERT-GFP localization to the Golgi region in uninfected cells (data not shown). In *C. trachomatis*-infected cells exposed to HPA-12, the patchy distribution of CERT-GFP was lost from the inclusion (Figure 6C, top row), although CERT-GFP was still present in the Golgi surrounding the inclusion, as evidenced by colocalization with p230, a *trans*-Golgi marker (Figure 6C, bottom row). These results indicate that ceramide binding and/or the transfer activity of CERT is required for its recruitment to the inclusion.

### SMS1 and SMS2 are recruited to distinct compartments at the inclusion

Since CERT cannot directly transfer SM [44], we considered the possibility that SMS1 or SMS2 are directly recruited to the inclusion, where they could synthesize SM following ceramide transfer to the inclusion by CERT. In uninfected cells, both SMS1 and SMS2 are found at the *trans*-Golgi while SMS2 also localizes to the plasma membrane (Figure S7 and [46], [69]).

**Figure 7:**
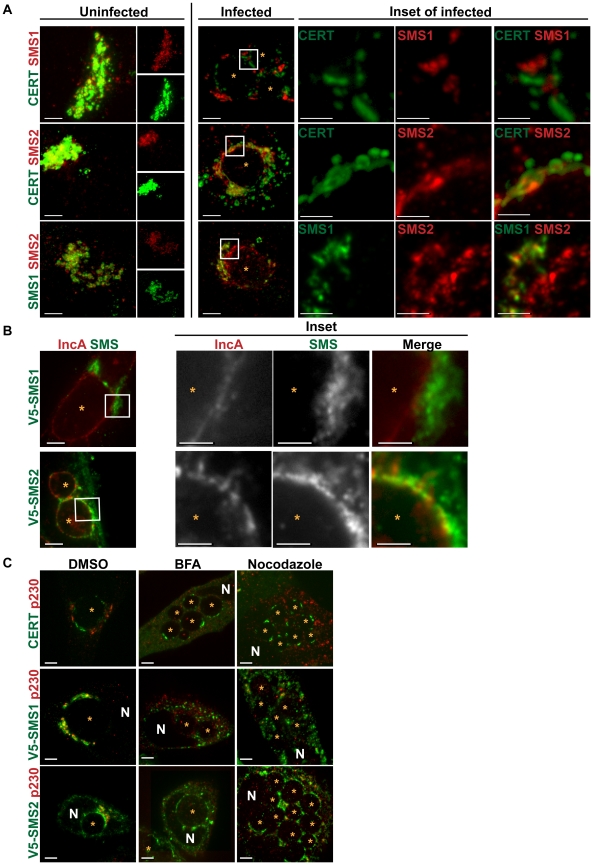
SMS1 and SMS2 localization in *C. trachomatis* infected cells. (A) HeLa cells co-transfected for 18 hrs with CERT-GFP and C-terminally 3xFLAG-tagged SMS1/SMS2 (upper 2 rows) or SMS1-V5 and C-terminally 3xFLAG-tagged SMS2 (bottom row) were infected with *C. trachomatis* L2 for 24 hrs, and then fixed and stained with antibodies to FLAG (red) and/or to V5 (green). Single channel images of uninfected cells are shown to the right. Enlargements of the boxed regions (inset) in infected samples are shown to the right of infected set. The exposure time for each filter set of all images was identical. Images shown are maximum intensity projections of confocal z-stacks (0.4-µm slices). CERT and SMS1 localization at the inclusion are distinct while CERT and SMS2 partially overlap. SMS2 localization partially overlaps with SMS1 around the inclusion, however SMS2 also localizes to the inclusion. At longer exposure times, SMS2 plasma membrane localization is evident. Scale bar = 5 µm, except the insets where scale bar = 2.5 µm. (B) HeLa cells transfected with SMS1-V5 (green) or SMS2-V5 (green) infected with *C. trachomatis* serovar D for 24 hrs and then fixed and stained with antibodies to IncA (red) to identify the inclusion membrane. Enlargements of the boxed regions (inset) are shown to the right. Images represent a single z slice from confocal images. The exposure time for each filter set for all images was identical. SMS2 but not SMS1 partially overlaps with IncA on the inclusion. Scale bar = 5 µm, except the insets where scale bar = 2.5 µm. (C) HeLa cells transfected for 18 hrs with CERT-GFP, SMS1-V5, or SMS2-V5 were infected with *C. trachomatis* L2, treated with 10 µM BFA or Nocodazole for 1–24 hpi, and then fixed and stained with antibodies to V5 (green) and to p230 (red) to identify the *trans*-Golgi. BFA and Nocodazole disrupted SMS1 localization around the inclusion but had no effect on SMS2 or CERT localization at the inclusion. Images represent a single z slice from confocal images. Scale bar = 5 µm. N, host nucleus; *, inclusion.

We examined the localization of CERT-GFP and C-terminally 3xFLAG-tagged SMS1/SMS2 or SMS1-V5 and C-terminally 3xFLAG-tagged SMS2 during *C. trachomatis* infection at 24 hpi. In uninfected cells, C-terminally 3xFLAG-tagged SMS1 and 3xFLAG-tagged SMS2 were located in close apposition to CERT-GFP at the Golgi (Figure 7A). In infected cells, C-terminally 3xFLAG-tagged SMS1 primarily localized to the fragmented Golgi surrounding the inclusion and displayed no overlap with CERT-GFP at the inclusion (Figure 7A). In contrast, C-terminally 3xFLAG-tagged SMS2 localized to both the fragmented Golgi as well as to the inclusion membrane, where it exhibited a distinct punctate pattern that partially overlapped with CERT-GFP (Figure 7A). Localization of SMS2 to the inclusion membrane was confirmed by co-staining infected cells expressing SMS2-V5 with antibodies to IncA; inclusion-membrane localized SMS2-V5 appeared to partially overlap with IncA whereas no overlap was seen between IncA and SMS1-V5 (Figure 7B).

To further distinguish between inclusion membrane versus Golgi localization, we examined the distribution of SMS1-V5 or SMS2-V5 in infected HeLa cells following disruption of the Golgi by BFA or by Nocodazole. As shown in Figure 7C, exposure of *C. trachomatis*-infected cells to either BFA or to Nocodazole caused SMS1-V5 to disperse throughout the cell while SMS2-V5 remained primarily associated with the inclusion, where a rim-like staining was observed. These results indicate that SMS2 is tethered to the inclusion during infection whereas SMS1 is primarily associated with the Golgi around the inclusion. In addition, SMS2 recruitment to the inclusion is independent of microtubules and of Arf1/GBF1-dependent vesicular trafficking.

The mechanism by which SMS1 or SMS2 localize to the *trans*-Golgi has not yet been elucidated. However, it was recently shown that SMS2 is palmitoylated at the carboxyl terminus and that this modification plays a role in targeting SMS2 to the plasma membrane [69]. We therefore tested whether SMS2 recruitment to the inclusion required palmitoylation by examining the localization of a C-terminally 3xFLAG-tagged SMS2 construct in which all four of the potential palmitoylation sites were mutated (C331, 332, 343, 348A) [69]. Although this mutant displays a decrease in PM distribution (data not shown and [69]), it was recruited to the inclusion in a similar fashion as C-terminally 3xFLAG-tagged SMS2 wild-type (Figure S7). This result suggests that palmitoylation is not required for SMS2 recruitment to the inclusion.

### SMS1 and SMS2 are required for *C. trachomatis* replication

**Figure 8:**
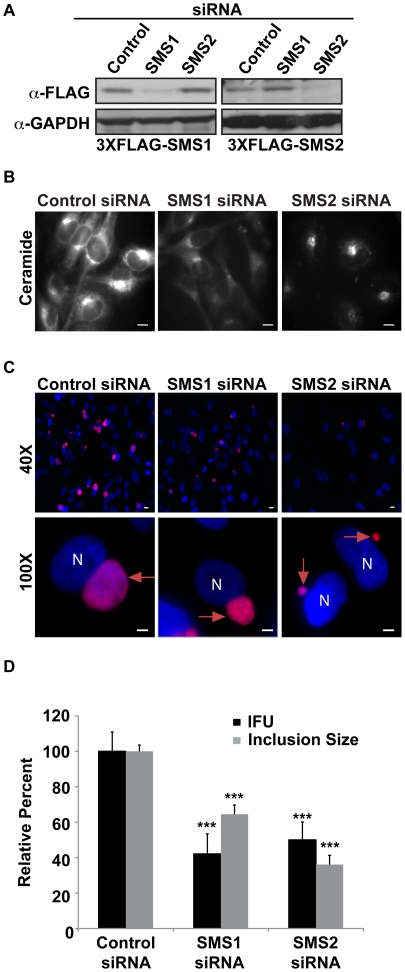
SMS1 and SMS2 contribute to *C. trachomatis* replication. (A) Western blot analysis of siRNA-treated samples. GAPDH was used as a loading control. (B) HeLa cells were depleted of SMS1 or SMS2 for 3 days and then labeled with BODIPY FL-Ceramide to analyze SM accumulation in the Golgi. The exposure time of all images was identical. SMS1 but not SMS2 depletion reduced BODIPY FL lipid accumulation in the Golgi. Scale bar = 5 µm. (C and D) HeLa cells were depleted of SMS1 or SMS2 for 3 days, infected with *C. trachomatis* L2 for 24 hrs, and then fixed and stained with an antibody to MOMP (red). Bacteria and host DNA were detected using DAPI (blue). (D) SMS1 and SMS2-depleted cells were analyzed for inclusion size and progeny formation. Values (mean ± standard error) are shown as percentage of control siRNA samples. SMS1 and SMS2 depletion reduced inclusion size and production of infectious progeny. Data are representative of 2 independent experiments. ***p<0.001, all samples compared to control siRNA treatment (ANOVA). N, host nucleus; red arrows point to inclusions. IFU, inclusion forming units. Scale bar = 5 µm.

Both SMS1 and SMS2 are required for host SM production, however their relative contributions to subcellular SM levels are distinct, with SMS1 generating the bulk of SM at the Golgi while SMS2 is responsible for SM biosynthesis at the plasma membrane [46], [56]. Our observation that SMS2 but not SMS1 is recruited to the inclusion membrane prompted us to determine their relative contributions to infection. Cells were depleted of SMS1 or SMS2 by siRNA [56] for 72 hrs and subsequently infected with *C. trachomatis* for 24 hrs. The efficiency of depletion was verified by western blot analysis in cells expressing C-terminally 3xFLAG-tagged SMS1 and 3xFLAG-tagged SMS2 (Figure 8A). Loss of SMS1 activity was confirmed by demonstrating decreased accumulation of BODIPY FL-Ceramide and BODIPY FL-SM at the Golgi apparatus (Figure 8B) [56]. Depletion of either SMS1 or SMS2 resulted in a significant reduction in relative inclusion size and progeny formation (Figures 8C–D). These results demonstrate that both SMS1 and SMS2 contribute to *C. trachomatis* replication and inclusion growth. Our results support the hypothesis that at least a portion of SM acquired by *C. trachomatis* may be directly synthesized on the inclusion membrane. In addition, SM synthesized at the nearby Golgi by SMS1 and SMS2 transported by a BFA-insensitive pathway may also contribute to replication.

## Discussion

Different pathogens have evolved a variety of unique strategies to establish a protective intracellular niche in which to replicate and to obtain essential nutrients from the host. For example, *Legionella pneumophila* replicates within a vacuole that is closely associated with the ER, while *Salmonella* and *Mycobacteria* species replicate within compartments that have characteristics of endosomes [70]. *Chlamydiae* are among the few known pathogens that occupy an exocytic compartment from which they acquire host-derived nutrients, including SM and cholesterol [6], [9], [10], [11], [12], [13], [14]. While previous work supports a role for canonical vesicular trafficking in the acquisition of SM by *Chlamydia* from the host, it has remained a mystery as to why inhibitors of vesicular trafficking have no effect on *Chlamydia* replication, given that host SM biosynthesis is necessary for bacterial replication [6], [12]. Here, we demonstrate that *C. trachomatis* co-opts key proteins involved in both vesicular and non-vesicular lipid trafficking pathways to acquire SM for distinct roles during infection, providing an intriguing explanation for this apparent paradox. We found that *C. trachomatis* recruits CERT, its ER binding partner VAP-A, and SM synthases to establish an on-site SM biosynthetic factory at or near the inclusion that is critical for *C. trachomatis* replication. In addition, we show that *C. trachomatis* co-opts the function of GBF1, a regulator of Arf1-dependent vesicular trafficking within the early secretory pathway, to further provide SM. This source of SM contributes to inclusion membrane growth and stability but is not essential for bacterial replication.

**Figure 9:**
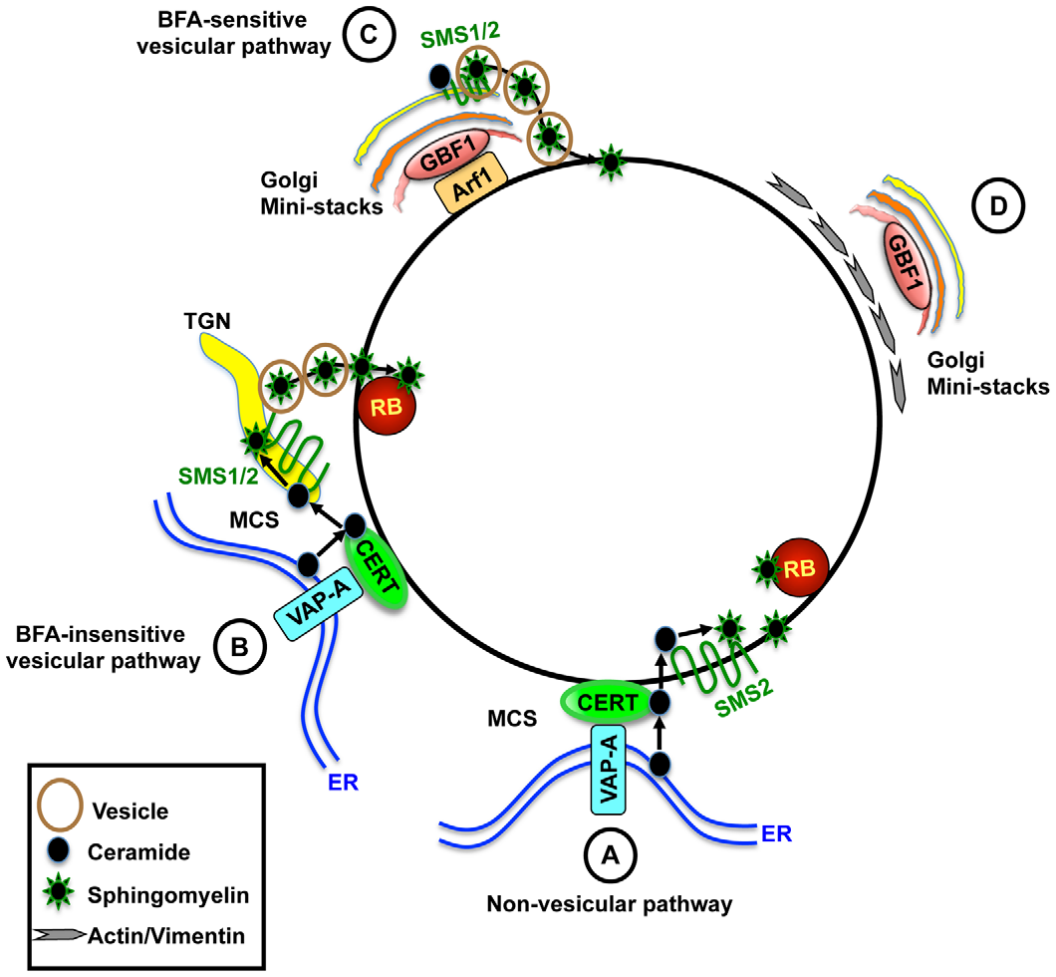
Model for the role of CERT and GBF1 in *C. trachomatis* infection. (A) CERT and VAP-A are recruited to the inclusion. CERT transfers ceramide from the ER to the inclusion. Inclusion membrane localized SMS2 converts ceramide to SM, which accumulates on the inclusion membrane and on RBs. (B) Alternatively, or in addition, CERT promotes recruitment of ER-Golgi MCS to the vicinity of the inclusion, promoting efficient SM synthesis near the inclusion. SM is then transferred to the inclusion by a BFA-insensitive pathway. (C) GBF1-dependent activation of Arf1 mediates vesicular trafficking of SM-containing vesicles to the inclusion for membrane growth and stability. (D) GBF1 activity contributes to the arrangement of the actin and vimentin networks around the inclusion for additional stability. CERT is essential for bacterial replication whereas GBF1 is important for inclusion membrane growth and stability. MCS, membrane contact site; TGN, trans-Golgi network.

We found that depletion or inhibition of CERT significantly impaired production of infectious progeny and SM acquisition. While these treatments decrease SM biosynthesis and would thus be expected to affect *C. trachomatis* replication, unexpectedly and most remarkably, we found that CERT was recruited to the inclusion membrane. Our results are consistent with two non-mutually exclusive mechanisms by which CERT could promote SM acquisition during infection (Figure 9). CERT could transport ceramide directly from the ER to the inclusion, where this lipid would serve as a substrate for inclusion membrane localized SMS2, allowing SM biosynthesis to proceed on the inclusion membrane (Figure 9, step A). SM would then be transferred from the inclusion membrane to intracellular RBs. Alternatively, or in addition, by virtue of its ability to bind to the ER and to the Golgi, CERT could help to coordinate recruitment of these organelles to the inclusion (Figure 9, step B). This process would bring the *trans*-Golgi localized SMS1 and SMS2 in close proximity to the ER and to the inclusion, thereby promoting efficient SM synthesis in the vicinity of the inclusion. Since CERT cannot transport SM [44], it is likely that this source of SM is subsequently transferred to the inclusion by a BFA-insensitive vesicular trafficking pathway. It is also possible that SM could be transferred from the Golgi at MCS to the inclusion by one of several mechanisms involving non-vesicular lipid exchange between membranes, such as transient hemifusion and/or stochastic collision with the Golgi [71].

Our results suggest that both SMS1 and SMS2 contribute to infection. The involvement of SMS1 was not surprising, since this enzyme is responsible for the bulk of total host cell SM biosynthesis (60–80%) [46], [56]. However, our finding that SMS2 was localized to the inclusion membrane and played a role in intracellular growth was unexpected since SMS2 participates primarily in SM biosynthesis at the plasma membrane and plays a minor role in overall host cell SM biosynthesis (20–40%) [46], [56]. In contrast to SMS1, inclusion membrane localized SMS2 and CERT are not disrupted by BFA, GCA, or Nocodazole, which may explain why bacterial replication is not affected by these drugs. We note that it is difficult by current methods to demonstrate that SM is synthesized directly on the inclusion, since SM can also arrive via vesicular trafficking [46], [56]. We speculate that by preferentially recruiting SMS2 over SMS1 during infection, *C. trachomatis* ensures a source of SM for itself without placing a huge burden on the host's ability to synthesize SM since SMS1 would still be active at the Golgi. Future studies will be required to unravel how SMS2 is recruited to the inclusion membrane.

How is CERT, a multi-domain protein, recruited to the inclusion? Although the recruitment of CERT to Golgi and ER membranes requires the PH and FFAT domains, respectively [47], we found that only the ceramide binding and/or ceramide transfer activity of CERT is required for its recruitment to the inclusion. While CKIγ2-induced phosphorylation of CERT inhibits its activity, CERT was still recruited to the inclusion, presumably because CERT is still able to bind ceramide. On the other hand, exposure to HPA-12 prevented CERT recruitment to the inclusion, possibly through its known ability to inhibit interaction of the START domain with membranes *in vitro* [60]. An alternative scenario is that HPA-12 binding to CERT could alter its ability to bind PI4P, VAP-A, or potentially a bacterial factor at the inclusion membrane through steric hindrance or conformational change. We favor the hypothesis that CERT binds to ceramide at the ER, prior to its recruitment to the inclusion, and subsequently interacts with the inclusion by an as yet to be identified bacterial or host protein. It is also possible that a small amount of ceramide and/or VAP-A may initially be incorporated into the inclusion membrane from either the plasma membrane during endocytosis or by fusion with the ER or ER-derived vesicles containing ceramide, which would then promote CERT recruitment. These initial events could then set up an amplification mechanism for subsequent ceramide transfer and SM synthesis. Further studies are needed to determine whether CERT transfer activity or ceramide binding alone is necessary for its recruitment to the inclusion, and whether either of these functions cooperates with a bacterial protein present in the inclusion membrane.

It was surprising that PI4P binding was not required for CERT recruitment to the inclusion, given the recent observation that PI4P binding is necessary and sufficient to recruit the PH domain of OSBP [40]. However, it should be noted that those studies were performed with only the PH domain of OSBP whereas our studies were performed in the context of the entire CERT protein containing a single point mutation (G67E) that abolishes PI4P binding. It is likely that there are multiple localization signals for CERT recruitment to the inclusion.

The dispensable requirement of PI4P binding for CERT recruitment to the inclusion likely explains why inclusions still form in LY-A cells expressing CERT (G67E) and why CERT still localizes to the inclusion in these cells. It is worth noting that recombinant CERT (G67E) is still able to transfer ceramide in an *in vitro* assay [45], suggesting that although SM synthesis is reduced in the LY-A cell line due to the inability of CERT to bind the Golgi and transfer ceramide [43], [66], SM synthesis may proceed at the inclusion because this specialized compartment has access to ceramide via CERT and at least one SMS isoform.

While the role of CERT in ceramide transfer and SM biosynthesis is clearly established, we cannot rule out the possibility CERT may have other functions in host cells relevant to *Chlamydia* infection. Previous work has suggested that there is an intimate association of the ER or ER proteins with the chlamydial inclusion [57], [58]. It is possible that CERT and VAP-A together may be important for coordinating ER-inclusion membrane contact sites. These membrane contact sites could be important for the inclusion to obtain other essential lipids or nutrients for growth. Although loss of CERT function in individual cells has no effect on cell survival [43], [66], knockout of CERT in mice is embryonic lethal as a result of severe defects in both the ER and mitochondria function [72]. In addition, *D. melanogaster* flies lacking functional CERT have a short lifespan and show enhanced susceptibility to oxidative stress [73].

Our studies provide further insights into the mechanism of BFA-sensitive SM acquisition. While it was previously suggested that *Chlamydia* occupies a post Golgi compartment with the prediction that trafficking from the *trans*-Golgi would be essential, our results demonstrate that acquisition of SM by *Chlamydia* requires Arf1 activation within the *cis*-Golgi but not Arf1 activation within the *trans*-Golgi (Figure 9, step C), suggesting that *Chlamydia* is not necessarily co-opting the entire organelle. Loss of SM acquisition following inhibition of GBF1 function correlated with a loss of inclusion membrane integrity and alterations in the localization of actin and vimentin cytoskeletal components surrounding the inclusion (Figure 9, step D). Whether the loss of inclusion integrity is a direct or indirect consequence of the collapse of the closely associated cytoskeletal cage or whether it results from the loss of SM acquisition, as suggested by Beatty and coworkers [15], remains to be determined. Of note, GCA was not reported to affect the stability of serovar E inclusions [15]; however, this disparity may reflect strain differences.

We observed that GBF1 and Arf1 have little overlap at the inclusion membrane during infection even though they extensively colocalize at the Golgi surrounding the inclusion. This observation could reflect a transient and dynamic interaction at the inclusion. Alternatively, we favor the hypothesis that GBF1 activates Arf1 at the Golgi and then Arf1-vesicles containing SM traffic to and fuse with the inclusion. Under these circumstances, GBF1-dependent activation of Arf1 at the Golgi rather than at the inclusion would be important, which could explain why there is little colocalization of these proteins at the inclusion. Consistent with this notion, we found that inhibition of GBF1 activity resulted in a loss of inclusion localized Arf1, except at the region of abutting inclusion membranes. This finding suggests that GBF1 is required for Arf1 recruitment to the outer region of the inclusion but not for Arf1 maintenance at closely apposed inclusions. We are currently investigating the role of Arf1 at this region.

Recent studies have revealed that Arf1 and GBF1 also play important roles in lipid droplet biogenesis [74], [75]. As lipid droplets are translocated into the lumen of the inclusion [76], [77], interference with Arf1 and/or GBF1 function could also affect *Chlamydia* interaction with lipid droplets. Notably, GBF1 colocalizes with Lda3 (unpublished results), a chlamydial protein that localizes to lipid droplets [77].

*Chlamydia* now joins the growing list of pathogens that subvert GBF1 or Arf1 function to establish an intracellular replicative niche, including poliovirus, coxsackievirus, coronavirus, hepatitis C virus, and *Legionella pneumophila* [26]. In contrast to these pathogens where depletion or inhibition of GBF1 by BFA significantly impairs replication, our findings demonstrate that GBF1 is more important for establishing a stable intracellular niche in which *C. trachomatis* replicates. Both poliovirus and coxsackievirus encode a protein (3A) that binds to and co-opts GBF1 function, while *L. pneumophila* secretes into the cell RalF, an Arf GEF, that regulates Arf1 localization to the *L. pneumophila*-containing phagosome [78], [79]. Since GBF1 was not localized on the inclusion membrane, it is possible that GBF1 communicates indirectly with the inclusion through a variety of host proteins or lipids that have been shown to bind GBF1 and that localize at or near the inclusion, including Rab1 [80], PI4P [81], and the Golgi matrix proteins, golgin 84 and p115 [26]. It will be important to determine whether the alterations in the actin and vimentin cytoskeleton during infection are directly or indirectly modulated by GBF1.

In summary, we show that *C. trachomatis* hijacks components of both vesicular and non-vesicular lipid trafficking pathways for SM acquisition, and that the SM obtained from these pathways is utilized in different ways by the pathogen. We hypothesize that SM acquired by CERT-dependent transport of ceramide and subsequent conversion to SM is necessary for *C. trachomatis* replication whereas SM acquired by the GBF1-dependent pathway is essential for inclusion growth and stability. Together, these observations provide an intriguing explanation for why inhibition of vesicular trafficking alone fails to affect intracellular replication. Moreover, our results describe the first example of a bacterial pathogen to co-opt CERT and reveal a novel strategy by which this organism creates its own SM biosynthetic factory. This work has identified novel targets that may prove useful in combating *Chlamydia* infections.

## Materials and Methods

### Reagents

D609 was purchased from Calbiochem. Brefeldin A, Nocodazole, and Exo1 were purchased from Sigma. Golgicide A (GCA) was kindly provided by D. Haslam (Washington University School of Medicine, St. Louis, MO). BODIPY FL C5 Ceramide was obtained from Molecular Probes. Construction of Arf1-GFP, Arf1-mCherry, and GFP-GBF1 were previously described [82], [83]. CERT-GFP, CERT (D324A)-GFP, CERT (G67E)-GFP, HcRed-VAP-A, HA-CKIγ2, and HPA-12 were kind gifts from K. Hanada (National Institute of Infectious Disease, Tokyo) and have been previously described [45], [59]. C-terminally V5-tagged SMS1 and SMS2 constructs were kind gifts from J. Holthuis (Utrecht University) and have been described previously [46]. C-terminally 3xFLAG-tagged SMS1 and SMS2 constructs were kind gifts from M. Tani (Kyushu University) and have been previously described [69]. GFP-p58 was kindly provided by C. Roy (Yale University). Unless otherwise indicated, the following concentrations of inhibitors were used: 5 µM HPA-12, 25 µg/ml D609, 10 µM GCA, 10 µM BFA, 10 µM Nocodazole, and 50 µg/ml Exo1. Antibodies were obtained from the following sources: mouse anti-*Chlamydia* FITC conjugate (Meridian Diagnostics), goat anti-*C. trachomatis* MOMP (Cortex Biochem), mouse anti-GAPDH (Chemicon), mouse anti-GBF1 (BD Transduction Laboratories), rabbit anti-BIG1 (Santa Cruz), rabbit anti-BIG2 (Bethyl Laboratories, Inc.), chicken anti-CERT (Sigma), rabbit anti-14-3-3β (Santa Cruz), mouse anti-vimentin (Sigma), mouse anti-FLAG (Sigma), rabbit anti-V5 (Sigma), mouse anti-HA (Covance), rabbit anti-p58 (Sigma), mouse anti-ceramide IgM (Clone15B4), rabbit anti-calnexin (Cell Signaling), rabbit anti-goat IgG horseradish peroxidase (HRP) (Calbiochem), goat anti-rabbit IgG HRP (Amersham Biosciences), goat anti-mouse HRP (Amersham Biosciences), donkey anti-goat Alexa 594 (Molecular Probes), donkey anti-goat Alexa 488 (Molecular Probes), chicken anti-mouse 594 (Molecular Probes), goat anti-mouse IgG1 Alexa 594 (Molecular Probes), donkey anti-goat Alexa 488 (Molecular Probes), Texas red-conjugated donkey anti-chicken IgY (Jackson Laboratories), and donkey anti-rabbit Alexa 488 (Molecular Probes). Texas red-conjugated phalloidin was obtained from Molecular Probes. Mouse anti-IncA antibodies were kind gifts from T. Hackstadt (Rocky Mountain Laboratories), D. Rockey (Oregon State University), and G. Zhong (University of Texas Health Science Center at San Antonio). All siRNAs, including non-targeting negative controls, were obtained from Dharmacon: Human GBF1 (J-019783-06), human BIG1 (D-012207-02, D-012207-03), and human BIG2 (MQ-012208-01) and have been previously described [33], [84]. Human SMS1 and SMS2 siRNA sequences have been previously described [56]. Human CERT siRNA was performed using siGENOME SMARTpool.

### Cell culture and *C. trachomatis* propagation

HeLa 229 cells and L929 cells were obtained from ATCC and passaged as previously described [85]. LY-A and LY-A/hCERT cells were obtained from RIKEN BRC through the National Bio-Resource Project of MEXT, Japan and have been previously described [43], [66]. CHO cells were maintained at 37°C with 5% CO_2_ in Ham's F12 containing 10% fetal bovine serum (FBS). *C. trachomatis* serovar L2 (LGV 434) and D (UW3-Cx) were propagated in L929 cells. *C. trachomatis* EBs were harvested from infected cells and purified using a Renografin step-gradient as described [86]. Unless otherwise indicated, experiments were performed with serovar L2.

### Immunofluorescence studies

Cells were grown on glass coverslips in 24-well plates and infected at a multiplicity of infection (MOI) of approximately 1–5 with *C. trachomatis* as described in the text. For expression of tagged constructs, cells were transfected with the indicated plasmid constructs using Effectene (Invitrogen) for 18 hrs prior to infection, following manufacturer's instructions. Cells were fixed in 4% paraformaldehyde (PFA) or methanol and stained with the appropriate antibodies. For anti-GBF1, anti-14-3-3β, anti-MOMP, anti-HA, anti-ceramide, anti-calnexin, anti-p58, and anti-V5 staining, cells were permeabilized with 0.05% saponin/0.2% bovine serum albumun (BSA) in phosphate buffered saline (PBS) for 30 minutes and blocked with 0.2% BSA/PBS for 30 minutes. For anti-CERT staining, cells were permeabilized with 0.5% Triton X-100/PBS for 3 minutes and blocked with 1% BSA/PBS for 20 minutes. For phalloidin staining, cells were permeabilized with 0.2% Triton/PBS and blocked with 1% BSA/PBS for 1 hr. For anti-vimentin and anti-IncA staining, cells were post-fixed in methanol for 5 minutes and blocked in 2% BSA/PBS for 1 hr. Cells were incubated with the appropriate primary and fluorophore-tagged secondary antibodies for 1 hr each, followed by washing 3 times in PBS. Anti-FLAG staining was performed as previously described [69]. Coverslips were mounted in Vectashield mounting media containing DAPI (Vector Laboratories) to identify bacteria and host cell nuclei. Images were acquired under 20, 40 or 100× oil objective using a Nikon Eclipse TE2000-E fluorescence microscope with Simple PCI imaging software (Compix, Inc.) or acquired under 100× oil objective using Nikon TE2000-PFS spinning disk confocal inverted microscope with Nikon Elements software. For each set of experiments, the exposure time for each filter set for all images was identical. Images were processed with Adobe Photoshop CS, Nikon Elements, or Imaris Software.

### Immunoblot analysis

Cells were lysed in lysis buffer (50 mM Tris HCl, pH 7.5, 150 mM NaCl, 0.1% SDS, 1% Nonidet P-40, 1% sodium deoxycholate, 1 mM sodium orthovanadate, 1 mM sodium fluoride, 1 mM okadaic acid, and Complete protease inhibitors (Roche Diagnostics)). After centrifugation at 20,800× *g* for 5 minutes to remove cell debris, the supernatants were transferred to fresh tubes. Proteins were separated on 10% sodium dodecyl sulfate-polyacrylamide gel electrophoresis (SDS-PAGE) gels and transferred to 0.45-µm pore Trans-blot nitrocellulose membranes (BioRad Laboratories). Membranes were blocked with 5% milk and probed with the indicated antibodies. Proteins were detected by enhanced chemiluminescence (ECL) (Amersham Biosciences) according to the manufacturer's protocol.

### RNAi studies

HeLa cells grown in 6-well plates were transfected with specific or control siRNAs according to manufacturer's protocol. At 72 hrs post transfection, cells were infected with *C. trachomatis* for 1 hr and then incubated for an additional 24 hrs. Knockdown efficiency was measured by western blot analysis for endogenous protein, or in the case of SMS1 and SMS2, knockdown efficiency was determined using HeLa cells expressing C-terminally 3xFLAG-tagged SMS constructs as previously described [56]. Knockdown efficiency for SMS1 was also determined using BODIPY FL-Ceramide (see below).

### Quantitation of primary inclusion formation, inclusion size, inclusion membrane disruption, and production of infectious progeny

HeLa cells were incubated with the indicated siRNA for 3 days and subsequently infected with *C. trachomatis* (MOI of 1–5) for 24 hrs. For drug treated samples, HeLa cells were infected with *C. trachomatis* for 1 hr and then incubated for an additional 23 hrs in the presence of the indicated drug. To quantify primary inclusion formation, cells were fixed and processed by immunofluorescence and processed as described below. To quantify production of infectious progeny, infected cells were scraped into media, lysed with sonication, and 5-fold serial dilutions were used to infect fresh HeLa monolayers that had been plated on coverslips for 24 hrs. For assessment of drug effects on bacterial viability and internalization, progeny was harvested from DMSO treated samples and left untreated or treated with the various inhibitors during the 1 hr infection of fresh HeLa monolayers. Cells were then washed free of the inhibitor and incubated for an additional 24 hrs. Inclusions were visualized by immunofluorescence with goat anti-MOMP antibodies and donkey anti-goat Alexa 594 or mouse anti-*Chlamydia* FITC conjugate. Images were analyzed using MetaMorph software (Molecular Devices, Sunnyvale, CA) from a minimum of 10 fields per sample. The relative inclusion forming units (IFUs) were expressed as a percentage of control siRNA or DMSO controls. The data is presented as mean ± standard error. To quantify the relative size of inclusions, approximately 200–350 cells were counted from 7–10 fields, the inclusion area analyzed by Metamorph using the mean area function, and the data is normalized to the percent area of control siRNA or DMSO-treated cells. To quantify inclusion disruption, approximately 350 cells were scored for apparent breaks in the inclusion membrane by staining with 14-3-3β and for concomitant release of bacteria into the cytosol. The data is normalized to percentage of similarly scored control or DMSO-treated cells.

### Sphingomyelin accumulation assay

HeLa cells were infected with *C. trachomatis* for 21 hrs and then treated with the indicated drug for an additional 3 hrs. At 24 hpi, live cells were then incubated for 30 minutes in serum-free MEM containing 1 µM BODIPY FL-Ceramide (Molecular Probes) complexed with 0.034% defatted bovine serum albumin (dfBSA) in MEM at 4°C in the dark. Cells were washed 3 times with serum free MEM and back exchanged with MEM/0.34% dfBSA for 1 hr. Cells were immediately visualized by fluorescence microscopy under a 40× objective. To assess SM accumulation in the Golgi upon depletion of CERT or SMS1, BODIPY FL-Ceramide labeling was performed at 72 hrs post siRNA treatment and fluorescence intensity in the Golgi region of siRNA-treated samples was compared to control siRNA. To assess SM accumulation by the inclusion upon depletion and inhibition of GBF1 and BIGs, cells were treated with the appropriate siRNA for 72 hrs, infected with L2, and BODIPY FL-Ceramide labeling was performed at 24 hpi. For quantitation of SM accumulation in the inclusion, mean fluorescence intensity of the inclusion was determined by defining regions of interest within the inclusion and dividing by the mean fluorescence intensity of the nucleus, which remained constant with all drug treatments. At least 5 fields were examined, and values were obtained from a total of 10–15 inclusions.

### Statistical analysis

Data represented the mean ± standard error of *n* experiments. Statistical analysis was performed using the software program Instat. The significance between groups was determined by ANOVA. A *p* value less than 0.05 was considered to be statistically significant.

### Gene IDs

Arf1 (GeneID 375), GBF1 (GeneID 8729), BIG1 (GeneID 10565), BIG2 (GeneID 10564), CERT (GeneID 10087), VAP-A (GeneID 9218), SMS1 (GeneID 259230), and SMS2 (GeneID 166929).

## Supporting Information

Figure S1 **Inhibition or depletion of GBF1 but not BIGs reduces SM acquisition and inclusion size.** Quantitation of SM acquisition by the inclusion following (A) depletion of GBF1, BIG1, and/or BIG2 and (B) inhibition of GBF1 with 10 µM GCA or BFA. Values (mean ± standard error) are shown as percentage of mean fluorescence intensities relative to DMSO or control siRNA-treated samples. ***p<0.001 (ANOVA). Inhibition or depletion of GBF1 decreased the inclusion fluorescence intensity compared to DMSO or control siRNA-treated samples, respectively. (C) HeLa cells were depleted of GBF1, BIG1, and/or BIG2 for 3 days, infected with *C. trachomatis* L2 for 24 hrs, and then fixed and stained with an antibody to MOMP (green) to identify bacteria. Bacteria and host DNA were detected using DAPI (blue). The exposure time for each filter set for all images was identical. N, host nucleus; red arrows point to inclusion. Scale bar = 5 µm. (D) Quantitation of inclusion size in GBF1 and BIG1/2 depleted cells. Values (mean ± standard error) are shown as percentage of control siRNA samples. GBF1 depletion reduced inclusion size. Data are representative of 3 independent experiments. ***p<0.001, all samples compared to control siRNA treatment (ANOVA). IFU, inclusion forming units.
(TIF)

Figure S2 **Inhibition of GBF1 alters the actin and vimentin structures surrounding the inclusion.** (A) HeLa cells were infected with *C. trachomatis* L2, treated with 10 µM BFA or GCA for 1–24 hpi, and then fixed and stained with antibodies to 14-3-3β (green) to identify the inclusion membrane and GBF1 (red). Bacteria and host DNA were detected using DAPI (blue). The exposure time for each filter set for all images was identical. The inclusion membrane is discontinuous (white arrows) and bacteria are released into the cytoplasm upon exposure to GCA or BFA. Note the compact peri-nuclear localization of GBF1 upon GCA and BFA treatment. (B–C) HeLa cells were infected with *C. trachomatis* L2, treated with 10 µM GCA for 1–24 hpi, and then fixed and stained with antibodies to MOMP (green) to identify bacteria and (B) vimentin (red) or (C) with phalloidin to stain actin (red). The exposure time for each filter set for all images was identical. Images represent a single z slice from confocal images. White arrows point to a region around the inclusion that is devoid of vimentin or actin and where bacteria are released into the cytoplasm. N, host nucleus; *, inclusion. Scale bar = 5 µm.
(TIF)

Figure S3 **CERT (G67E) is recruited to inclusions in LY-A cells.** LY-A mutant cell line expressing CERT (G67E) or LY-A cell line complemented with wild type human CERT (LY-A/hCERT) were infected with *C. trachomatis* L2 for 24 hrs and then fixed and stained with antibodies to CERT (red) and MOMP (green). The exposure time for each filter set for all images was identical. Images shown are maximum intensity projections of confocal z-stacks (0.4-µm slices). *, inclusion; Scale bar = 5 µm.
(TIF)

Figure S4 **CERT recruitment to the inclusion is not dependent on GBF1 function.** (A) HeLa cells transfected with CERT-GFP were infected with *C. trachomatis* L2 for 2 hrs in the absence or presence of 10 µM GCA and stained with an antibody to the p58 (red), an ER-Golgi intermediate compartment marker. Bacteria and host DNA were detected using DAPI (blue). Enlargements of the boxed region (inset) are shown to the right. GCA did not prevent recruitment of CERT to the nascent inclusion. Scale bar = 5 µm, except in the inset, where scale bar = 2.5 µm. (B) HeLa cells transfected with CERT-GFP were left uninfected or infected with *C. trachomatis* L2 for 8 or 24 hrs in the presence of 10 µM GCA at 1–24 hpi. Images shown are maximum intensity projections of confocal z-stacks (0.4-µm slices). CERT-GFP recruitment to the inclusion was not dependent upon GBF1 function at mid or late times during infection. N, host nucleus. Red arrows point to inclusions. Scale bar = 5 µm. (C) HeLa cells co-expressing CERT-GFP and Arf1-RFP were left uninfected or infected with *C. trachomatis* L2 for 8 hrs. Images shown are maximum intensity projections of confocal z-stacks (0.4-µm slices). CERT-GFP localizes to both the Golgi and the inclusion during infection. N, host nucleus. Red arrows point to inclusions. Scale bar = 5 µm.
(TIF)

Figure S5 **Ceramide localizes on and around the inclusion.** (A) HeLa cells were infected with *C. trachomatis* serovar D and then fixed and stained with antibodies to ceramide (red) and IncA (green) to identify the inclusion membrane. (B–C) HeLa cells transfected with (B) Arf1-GFP or (C) CERT-GFP were infected with *C. trachomatis* L2, and then fixed and stained with antibodies to ceramide (red). Enlargements of the boxed regions (inset) are shown to the right. Images represent a single z slice from confocal images. Ceramide was localized to both the inclusion membrane as well as the region adjacent to the inclusion. *, inclusion. Scale bar, ∼5 µm.
(TIF)

Figure S6 **ER Localization in** ***C. trachomatis*** **-infected cells expressing CERT domain mutants.** HeLa cells were transfected with CERT-GFP, CERT (D324A)-GFP, or CERT (G67E)-GFP, infected with *C. trachomatis* L2 for 24 hrs, and then fixed and stained with antibodies to calnexin (red) to identify the ER. The exposure time for each filter set of all images was identical. Images shown are maximum intensity projections of confocal z-stacks (0.4-µm slices). The ER does not colocalize with wild type or mutant CERT at the inclusion. N, host nucleus; *, inclusion. Scale bar = 5 µm.
(TIF)

Figure S7 **Palmitoylation is not required for SMS2 recruitment to the inclusion.** HeLa cells transfected for 18 hrs with C-terminally 3xFLAG-tagged SMS1, 3xFLAG-tagged SMS2, or 3xFLAG-tagged SMS2 palmitoylation mutant (C331, 332, 343, 348A) were infected with *C. trachomatis* L2 for 24 hrs and then fixed and stained with anti-FLAG (red). Bacteria and host DNA were detected using DAPI (blue). The SMS2 palmitoylation mutant was recruited to the inclusion membrane. N, host nucleus; *, inclusion. Scale bar = 5 µm.
(TIF)

Video S1 **CERT-GFP distribution on** ***C. trachomatis*** **inclusions.** HeLa cells transfected for 18 hrs with CERT-GFP were infected with *C. trachomatis* L2 for 24 hrs. Confocal z-slices were taken every 0.4 µm and processed using IMARIS software to obtain a three dimensional (3D) reconstruction of the area. CERT-GFP decorates the inclusion membrane surface in a non-homogenous, patchy distribution.
(MOV)
