# Correction: *Chlamydia trachomatis* Co-opts GBF1 and CERT to Acquire Host Sphingomyelin for Distinct Roles during Intracellular Development

**DOI:** 10.1371/annotation/f8e7c7e3-c347-4243-9146-db77900cb90c

**Published:** 2013-08-06

**Authors:** Cherilyn A. Elwell, Shaobo Jiang, Jung Hwa Kim, Albert Lee, Torsten Wittmann, Kentaro Hanada, Paul Melancon, Joanne N. Engel

The following should be included as the first sentence of the abstract: 

“The strain designated Chlamydia trachomatis serovar L2 that was used for experiments in this paper is Chlamydia muridarum, a species closely related to C. trachomatis (and formerly termed the Mouse Pneumonitis strain of C. trachomatis). This conclusion was verified by deep sequencing and by PCR using species-specific primers. All data presented in the results section that refer to C. trachomatis should be interpreted as referring to C. muridarum. The authors have confirmed the identity of the C. trachomatis serovar D strain used in this study by PCR and sequencing of the ompA gene.”

