# Supplementary figures and images for: Chlamydia trachomatis Co-opts GBF1 and CERT to Acquire Host Sphingomyelin for Distinct Roles during Intracellular Development
Source: PLoS Pathog. 2011 Sep 1;7(9):e1002198. doi: 10.1371/journal.ppat.1002198 (PMC3164637; doi:10.1371/journal.ppat.1002198)

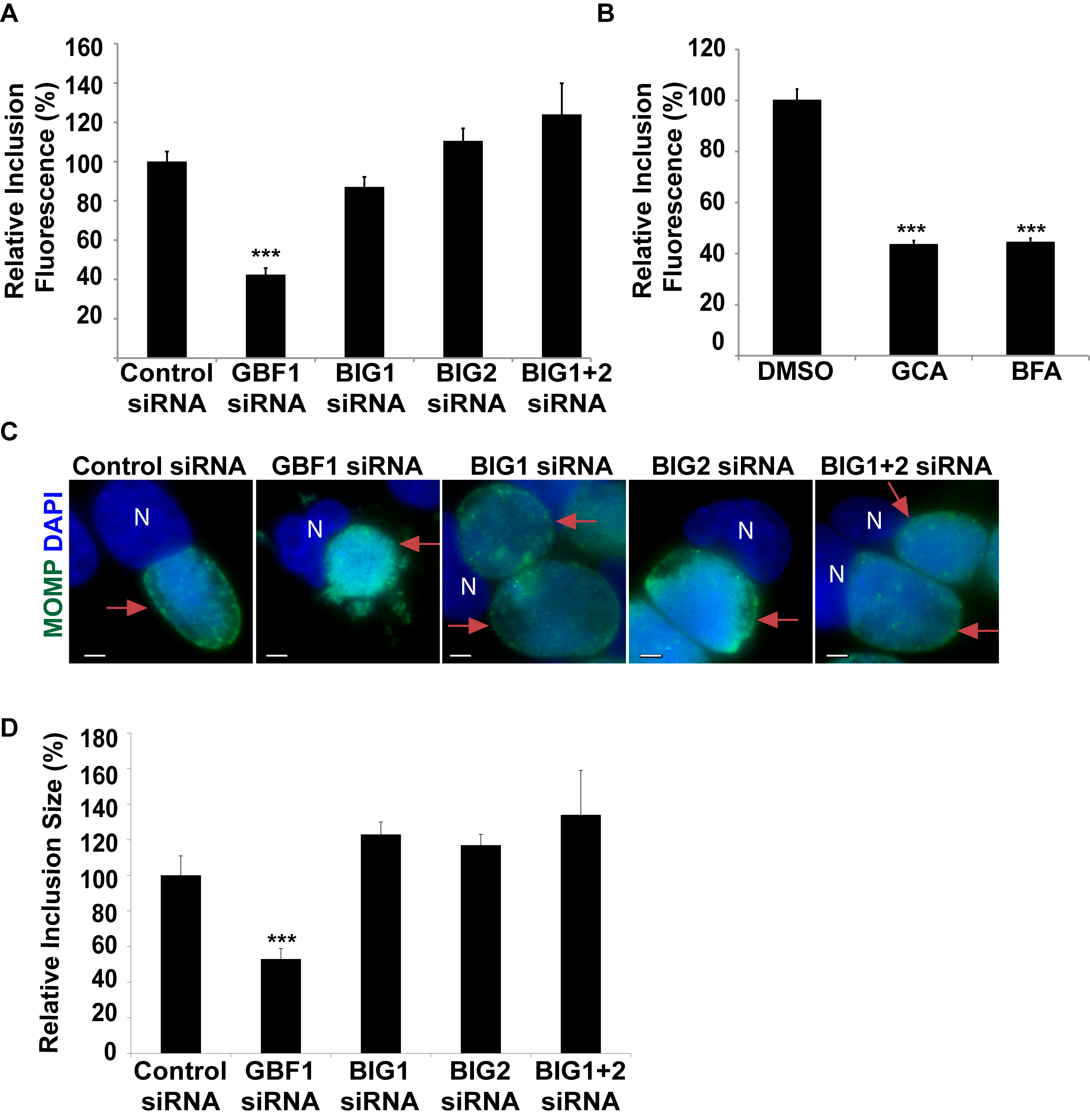

Supplement: Figure S1 — Inhibition or depletion of GBF1 but not BIGs reduces SM acquisition and inclusion size. Quantitation of SM acquisition by the inclusion following (A) depletion of GBF1, BIG1, and/or BIG2 and (B) inhibition of GBF1 with 10 µM GCA or BFA. Values (mean ± standard error) are shown as percentage of mean fluorescence intensities relative to DMSO or control siRNA-treated samples. ***p<0.001 (ANOVA). Inhibition or depletion of GBF1 decreased the inclusion fluorescence intensity compared to DMSO or control siRNA-treated samples, respectively. (C) HeLa cells were depleted of GBF1, BIG1, and/or BIG2 for 3 days, infected with C. trachomatis L2 for 24 hrs, and then fixed and stained with an antibody to MOMP (green) to identify bacteria. Bacteria and host DNA were detected using DAPI (blue). The exposure time for each filter set for all images was identical. N, host nucleus; red arrows point to inclusion. Scale bar = 5 µm. (D) Quantitation of inclusion size in GBF1 and BIG1/2 depleted cells. Values (mean ± standard error) are shown as percentage of control siRNA samples. GBF1 depletion reduced inclusion size. Data are representative of 3 independent experiments. ***p<0.001, all samples compared to control siRNA treatment (ANOVA). IFU, inclusion forming units. (TIF) [file ppat.1002198.s001.tif]

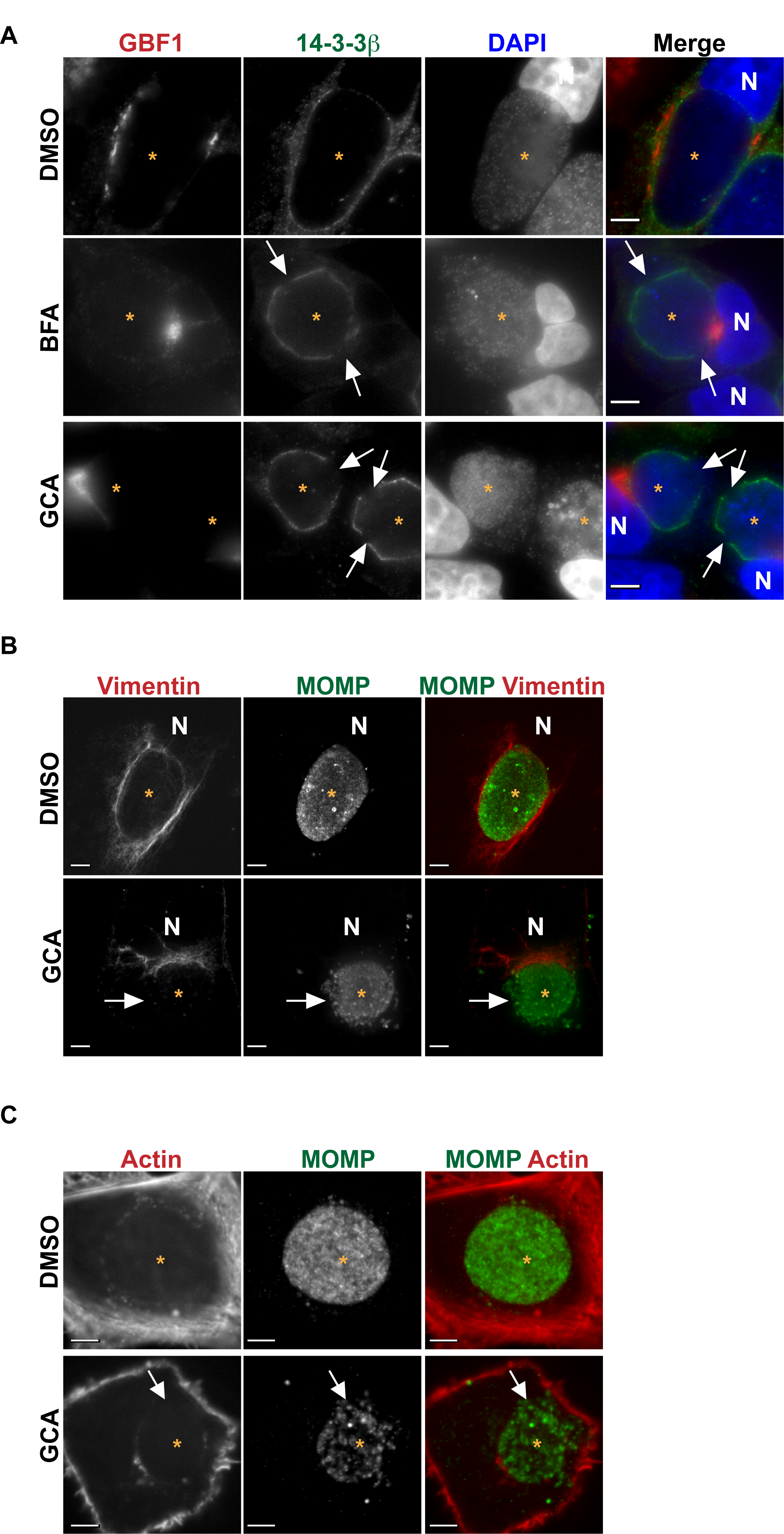

Supplement: Figure S2 — Inhibition of GBF1 alters the actin and vimentin structures surrounding the inclusion. (A) HeLa cells were infected with C. trachomatis L2, treated with 10 µM BFA or GCA for 1–24 hpi, and then fixed and stained with antibodies to 14-3-3β (green) to identify the inclusion membrane and GBF1 (red). Bacteria and host DNA were detected using DAPI (blue). The exposure time for each filter set for all images was identical. The inclusion membrane is discontinuous (white arrows) and bacteria are released into the cytoplasm upon exposure to GCA or BFA. Note the compact peri-nuclear localization of GBF1 upon GCA and BFA treatment. (B–C) HeLa cells were infected with C. trachomatis L2, treated with 10 µM GCA for 1–24 hpi, and then fixed and stained with antibodies to MOMP (green) to identify bacteria and (B) vimentin (red) or (C) with phalloidin to stain actin (red). The exposure time for each filter set for all images was identical. Images represent a single z slice from confocal images. White arrows point to a region around the inclusion that is devoid of vimentin or actin and where bacteria are released into the cytoplasm. N, host nucleus; *, inclusion. Scale bar = 5 µm. (TIF) [file ppat.1002198.s002.tif]

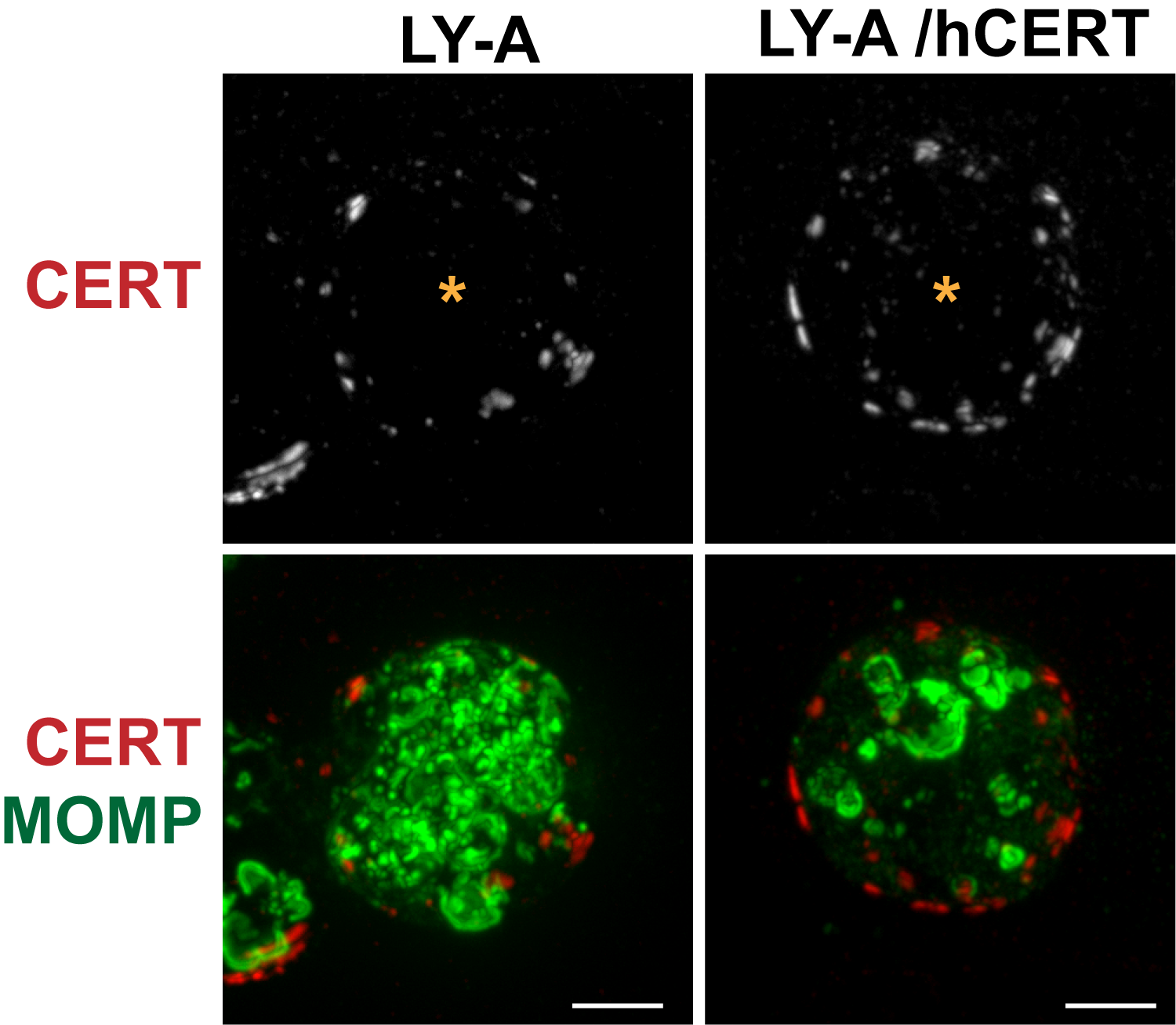

Supplement: Figure S3 — CERT (G67E) is recruited to inclusions in LY-A cells. LY-A mutant cell line expressing CERT (G67E) or LY-A cell line complemented with wild type human CERT (LY-A/hCERT) were infected with C. trachomatis L2 for 24 hrs and then fixed and stained with antibodies to CERT (red) and MOMP (green). The exposure time for each filter set for all images was identical. Images shown are maximum intensity projections of confocal z-stacks (0.4-µm slices). *, inclusion; Scale bar = 5 µm. (TIF) [file ppat.1002198.s003.tif]

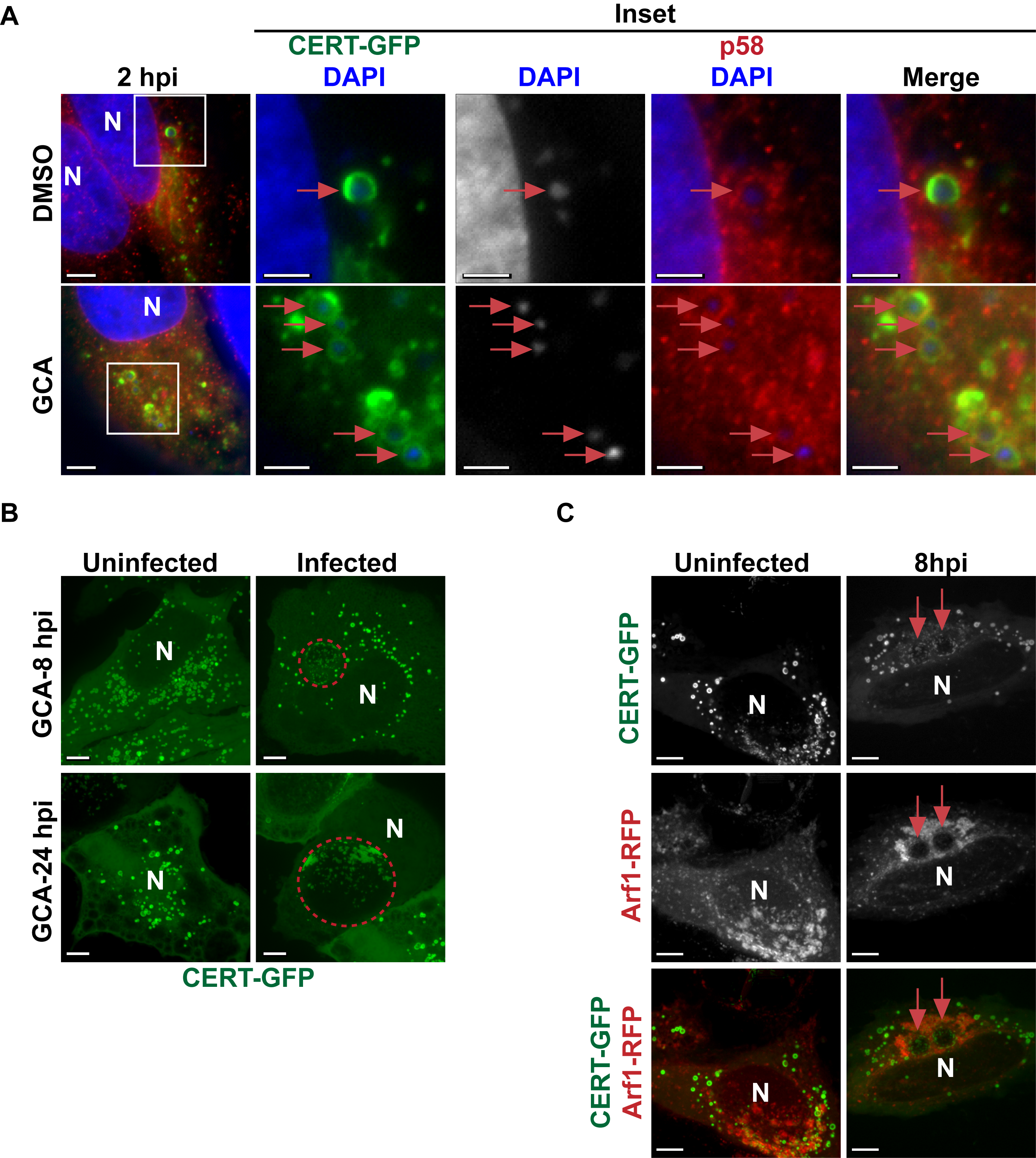

Supplement: Figure S4 — CERT recruitment to the inclusion is not dependent on GBF1 function. (A) HeLa cells transfected with CERT-GFP were infected with C. trachomatis L2 for 2 hrs in the absence or presence of 10 µM GCA and stained with an antibody to the p58 (red), an ER-Golgi intermediate compartment marker. Bacteria and host DNA were detected using DAPI (blue). Enlargements of the boxed region (inset) are shown to the right. GCA did not prevent recruitment of CERT to the nascent inclusion. Scale bar = 5 µm, except in the inset, where scale bar = 2.5 µm. (B) HeLa cells transfected with CERT-GFP were left uninfected or infected with C. trachomatis L2 for 8 or 24 hrs in the presence of 10 µM GCA at 1–24 hpi. Images shown are maximum intensity projections of confocal z-stacks (0.4-µm slices). CERT-GFP recruitment to the inclusion was not dependent upon GBF1 function at mid or late times during infection. N, host nucleus. Red arrows point to inclusions. Scale bar = 5 µm. (C) HeLa cells co-expressing CERT-GFP and Arf1-RFP were left uninfected or infected with C. trachomatis L2 for 8 hrs. Images shown are maximum intensity projections of confocal z-stacks (0.4-µm slices). CERT-GFP localizes to both the Golgi and the inclusion during infection. N, host nucleus. Red arrows point to inclusions. Scale bar = 5 µm. (TIF) [file ppat.1002198.s004.tif]

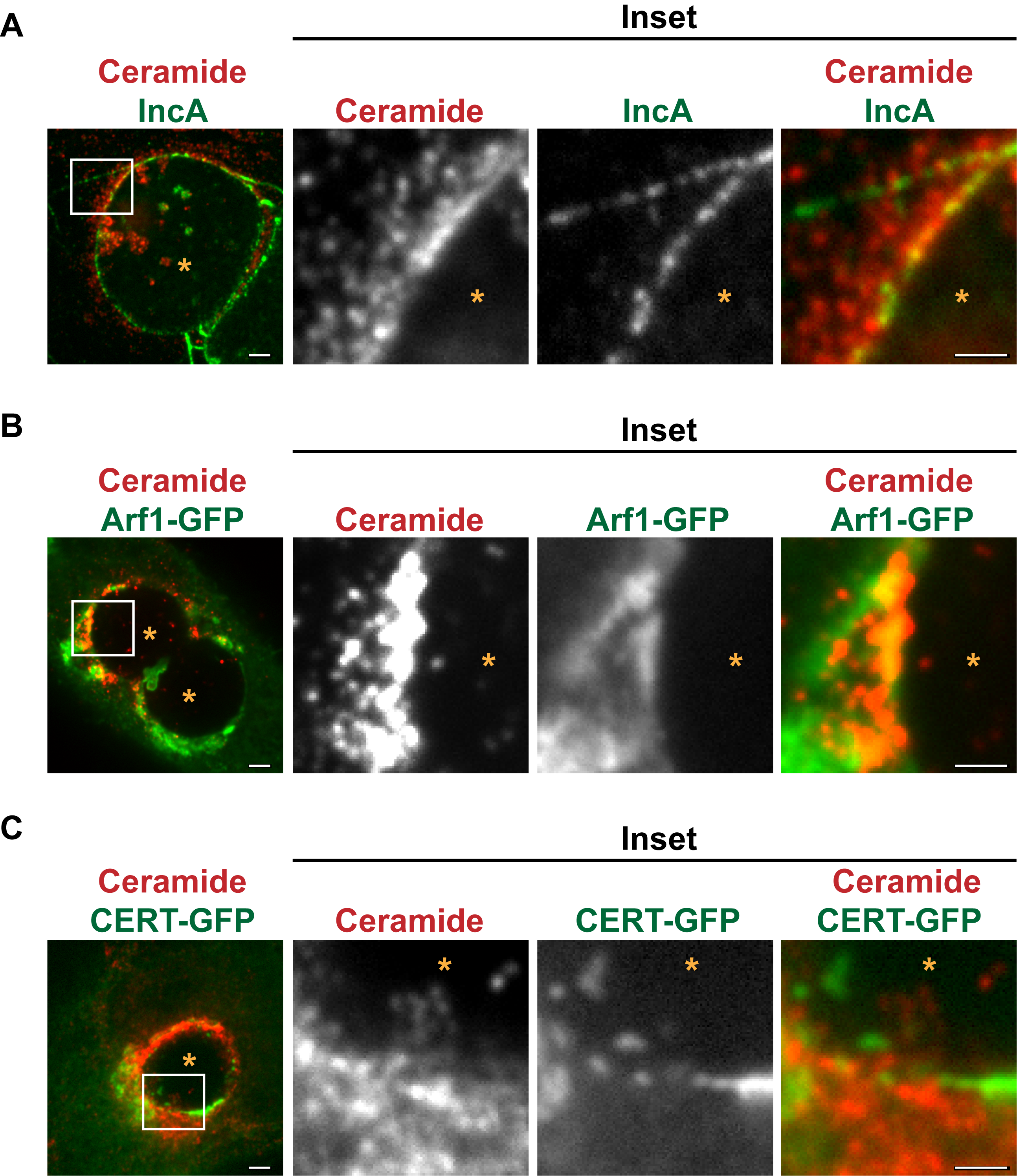

Supplement: Figure S5 — Ceramide localizes on and around the inclusion. (A) HeLa cells were infected with C. trachomatis serovar D and then fixed and stained with antibodies to ceramide (red) and IncA (green) to identify the inclusion membrane. (B–C) HeLa cells transfected with (B) Arf1-GFP or (C) CERT-GFP were infected with C. trachomatis L2, and then fixed and stained with antibodies to ceramide (red). Enlargements of the boxed regions (inset) are shown to the right. Images represent a single z slice from confocal images. Ceramide was localized to both the inclusion membrane as well as the region adjacent to the inclusion. *, inclusion. Scale bar, ∼5 µm. (TIF) [file ppat.1002198.s005.tif]

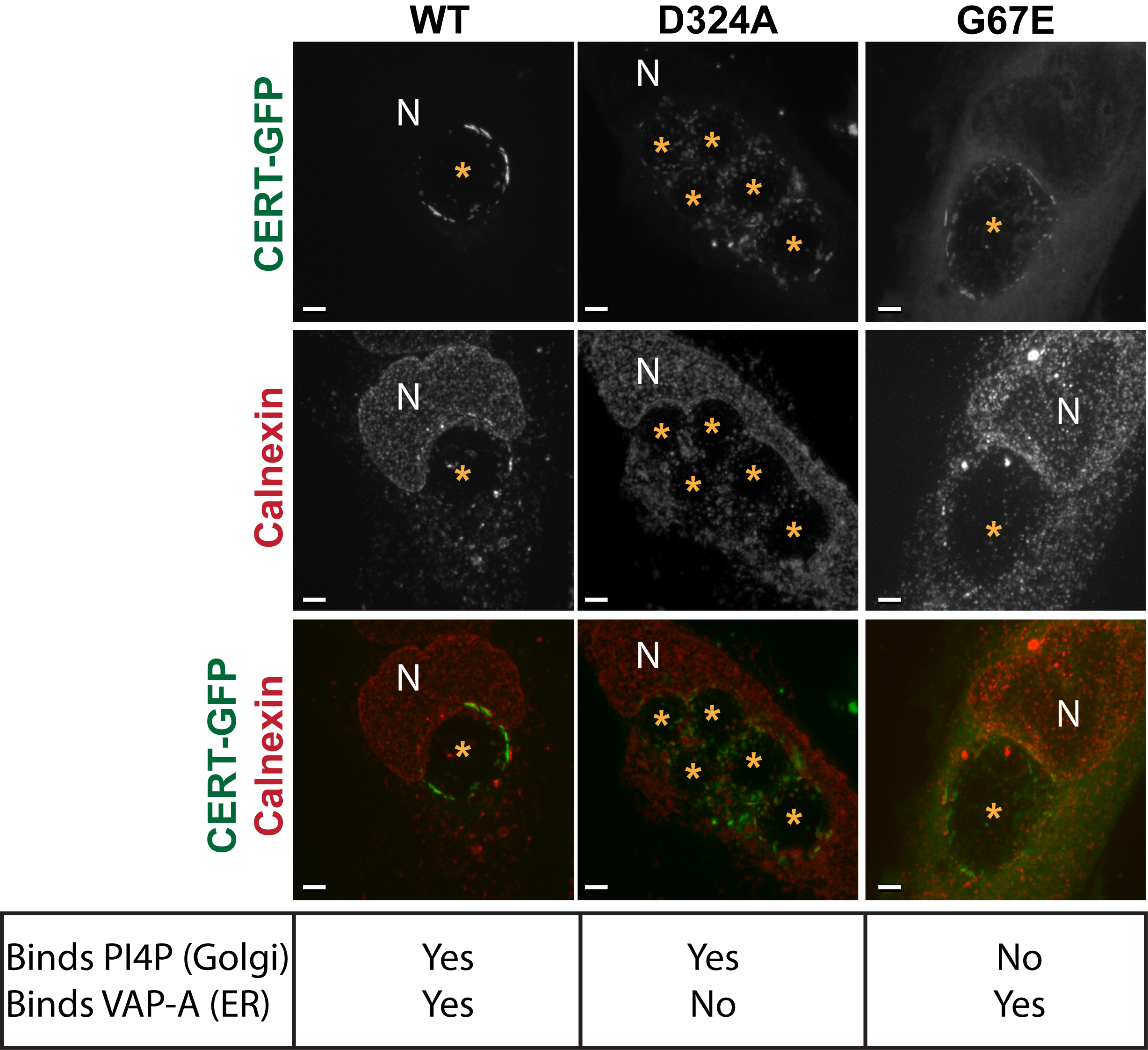

Supplement: Figure S6 — ER Localization in C. trachomatis -infected cells expressing CERT domain mutants. HeLa cells were transfected with CERT-GFP, CERT (D324A)-GFP, or CERT (G67E)-GFP, infected with C. trachomatis L2 for 24 hrs, and then fixed and stained with antibodies to calnexin (red) to identify the ER. The exposure time for each filter set of all images was identical. Images shown are maximum intensity projections of confocal z-stacks (0.4-µm slices). The ER does not colocalize with wild type or mutant CERT at the inclusion. N, host nucleus; *, inclusion. Scale bar = 5 µm. (TIF) [file ppat.1002198.s006.tif]

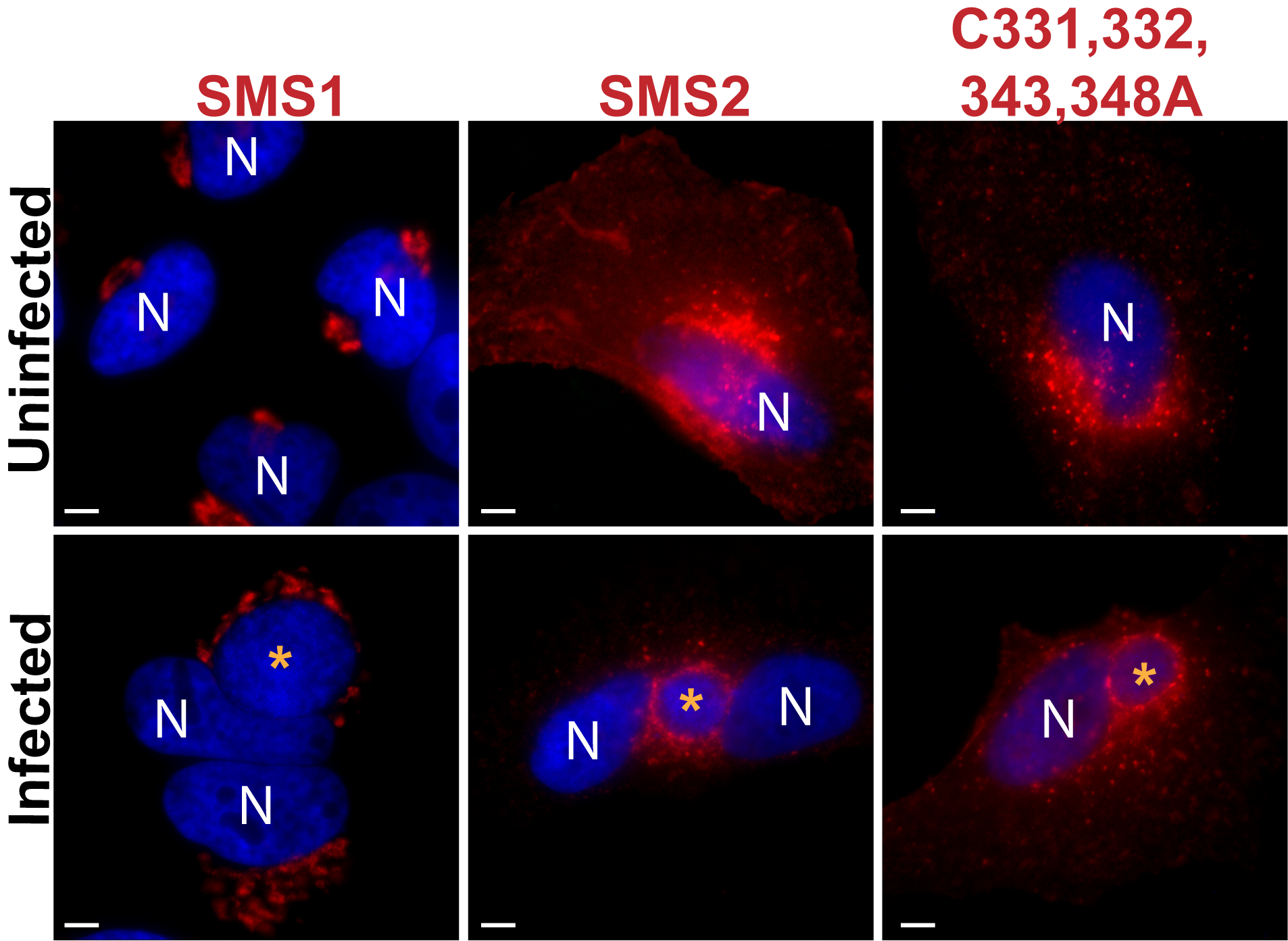

Supplement: Figure S7 — Palmitoylation is not required for SMS2 recruitment to the inclusion. HeLa cells transfected for 18 hrs with C-terminally 3xFLAG-tagged SMS1, 3xFLAG-tagged SMS2, or 3xFLAG-tagged SMS2 palmitoylation mutant (C331, 332, 343, 348A) were infected with C. trachomatis L2 for 24 hrs and then fixed and stained with anti-FLAG (red). Bacteria and host DNA were detected using DAPI (blue). The SMS2 palmitoylation mutant was recruited to the inclusion membrane. N, host nucleus; *, inclusion. Scale bar = 5 µm. (TIF) [file ppat.1002198.s007.tif]
